# One- and two-electron reduction of triarylborane-based helical donor–acceptor compounds†‡

**DOI:** 10.1039/d1sc02409d

**Published:** 2021-07-27

**Authors:** Xiangqing Jia, Jörn Nitsch, Zhu Wu, Alexandra Friedrich, Johannes Krebs, Ivo Krummenacher, Felipe Fantuzzi, Holger Braunschweig, Michael Moos, Christoph Lambert, Bernd Engels, Todd B. Marder

**Affiliations:** Institut für Anorganische Chemie and Institute for Sustainable Chemistry & Catalysis with Boron, Julius-Maximilians-Universität Würzburg Am Hubland 97074 Würzburg Germany todd.marder@uni-wuerzburg.de; Institut für Organische Chemie, Julius-Maximilians-Universität Würzburg Am Hubland 97074 Würzburg Germany; Institut für Physikalische und Theoretische Chemie, Julius-Maximilians-Universität Würzburg Am Hubland 97074 Würzburg Germany

## Abstract

One-electron chemical reduction of 10-(dimesitylboryl)-*N*,*N*-di-*p*-tolylbenzo[*c*]phenanthrene-4-amine (3-B(Mes)_2_-[4]helix-9-N(*p*-Tol)_2_) **1** and 13-(dimesitylboryl)-*N*,*N*-di-*p*-tolyldibenzo[*c*,*g*]phenanthrene-8-amine (3-B(Mes)_2_-[5]helix-12-N(*p*-Tol)_2_) **2** gives rise to monoanions with extensive delocalization over the annulated helicene rings and the boron p_*z*_ orbital. Two-electron chemical reduction of **1** and **2** produces open-shell biradicaloid dianions with temperature-dependent population of the triplet states due to small singlet-triplet gaps. These results have been confirmed by single-crystal X-ray diffraction, EPR and UV/vis-NIR spectroscopy, and DFT calculations.

## Abstract 2

Stepwise chemical reduction of D–π–A triarylborane-based helicenes gives the corresponding monoanions and dianions with delocalized unpaired electrons. The structures were confirmed by single crystal X-ray diffraction.

## Graphical abstract

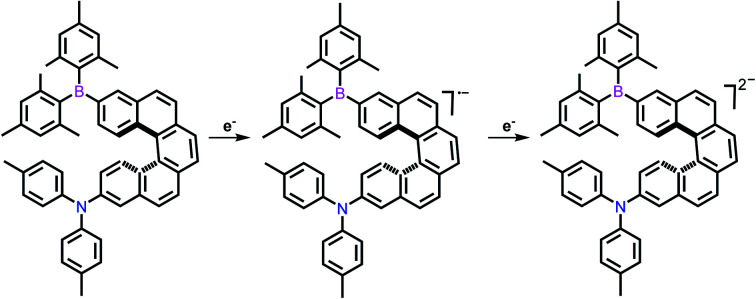

## Introduction

Three-coordinate organoboron compounds have attracted much attention for various optical and optoelectronic applications.^1^ Because of its empty p_*z*_ orbital, the boron functions as a strong electron acceptor and spin carrier. Isolation of stable boron-containing radicals is important both in fundamental chemistry and in practical applications, such as chemical sensors, organic synthesis and polymerizations.^2^ Consequently, investigation of the spin density distribution associated with borane radicals has attracted increasing interest.^2*b*,2*d*,3^ Stabilized by delocalization of the unpaired electron on two or more boron atoms, a number of radicals of diboryl compounds and polyborane clusters have been isolated.^4^ However, mononuclear boron radicals are still relatively rare. Reduction of triarylboranes has been studied since the 1920s,^5^ in systems in which the boron radical was stabilized by steric shielding to prevent bimolecular reactivity or coupled with spin delocalization *via* π-conjugation.

Spin-delocalization is an inherent feature of π-conjugated open-shell molecules containing one or more unpaired electrons.^6^ Helicenes are *ortho*-fused polycyclic aromatic hydrocarbons with nonplanar distorted molecular orbitals.^7^ Spin-delocalization through helical backbones adds an interesting element of chirality. The unique inherent optical and magnetic properties resulting from unpaired electron spin delocalization within helical π-systems are of fundamental interest to the design and development of organic optoelectronic materials and devices.

Recently, we reported the synthesis, photophysical, and electronic properties of triarylborane-based helical donor and acceptor compounds (3-B(Mes)_2_-[4]helix-9-N(*p*-Tol)_2_) **1** and (3-B(Mes)_2_-[5]helix-12-N(*p*-Tol)_2_) **2**.^8^ The donor- and acceptor-substituted nonplanar aromatic rigid helicene derivatives exhibit moderate to high fluorescence quantum yields in solution, and up to 1.00 in the solid state, which are higher than those reported for the unsubstituted helicenes.^9^ Such D–π–A compounds possess large electric dipole moments in the excited state, exhibiting strong intramolecular charge-transfer (ICT) emission, which make them attractive for various applications.^1*n*,10^ Zhao and co-workers also disclosed a triarylborane-based [5]helicene with enhancement of the fluorescence efficiency or full-color circularly polarized luminescence.^11^ Herein, we report the stepwise chemical reduction of the D-helix-A compounds **1** and **2** to produce their corresponding monoanions and dianions. The structures of products were confirmed by single crystal X-ray diffraction. We also investigated the reduced monoanion and dianion species by EPR spectroscopy and theoretical calculations.

## Results and discussion

We previously reported cyclic voltammetry studies of **1** and **2**, which revealed two reversible reductions at −2.29, −2.72 V (*vs.* Fc/Fc^+^) and −2.29, −2.71 V (*vs.* Fc/Fc^+^), respectively, in THF with [*n*Bu_4_N][PF_6_] as the supporting electrolyte.^8^ The appearance of two reversible reduction peaks suggested that their singly and doubly reduced species might be stable enough for isolation. We therefore carried out the stepwise chemical reduction of **1** and **2** to isolate their respective monoanions and dianions, and to study their structural and electronic properties. Compounds **1** and **2** also show similar reversible oxidations, indicating that the oxidized species might also be stable enough for isolation. However, we could not isolate the radical cations of either **1** or **2**, possibly due to subsequent reaction of the triarylamine radical, as observed in earlier studies.^12^

**Scheme 1:**
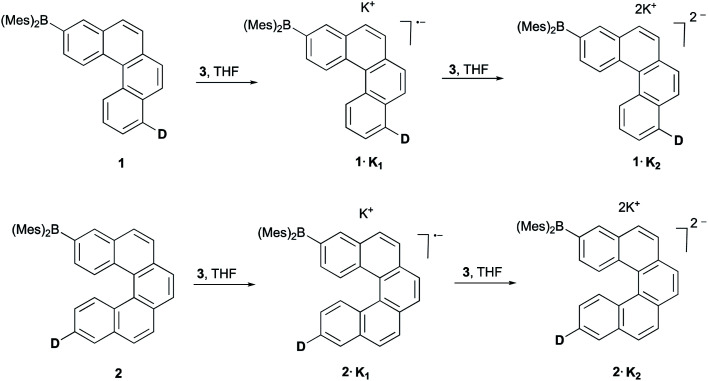
Stepwise reduction of **1** and **2** using [K(18-crown-6)(THF)_2_] naphthalenide (**3**) in THF to give rise to the corresponding monoanions and dianions, **D** = di-*p*-tolylamino.

The reduction of **1** and **2** with 1.0 equiv. of [K(18-crown-6)(THF)_2_] naphthalenide (**3**) in THF at room temperature afforded dark red solutions. Upon slow diffusion of dry *n*-pentane into the reaction mixture, dark red crystals of the radical monoanion salts **1·K1** and **2·K1** were obtained in 76% and 70% yields, respectively. Upon addition of 2.5 equiv. of **3** in THF, the color of the solution changed to dark blue. By slow diffusion of dry *n*-pentane into the reaction mixture, dark blue crystals of the dianion salt **1·K2** and a disordered 1 : 1 co-crystal of **2·K1** and **2·K2** were obtained in 60% and 68% yields, respectively (Scheme 1).

**Fig. 1:**
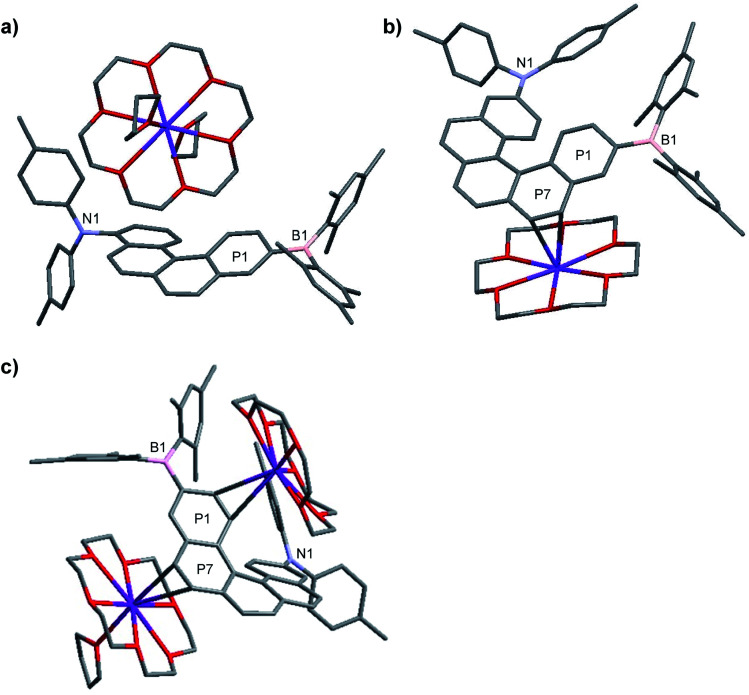
Molecular structures of racemic **1·K1** (a), **2·K1** (b) and **2·K1**/**2·K2** (c) in the solid state at 100 K. Hydrogen atoms and solvent molecules (THF) in **1·K1** are omitted for clarity. For **2·K1**/**2·K2**, the B(Mes)_2_ group and the upper 18-crown-6 with potassium ion are disordered. P1 and P7 refer to the planes of the arene rings.

**Fig. 2:**
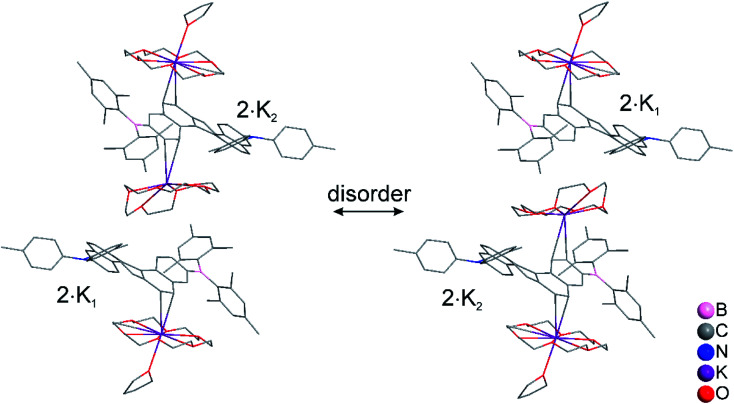
Molecular structure of the racemic, disordered 1 : 1 co-crystal of **2·K1** and **2·K2**, in the solid state at 100 K. Hydrogen atoms are omitted for clarity. Only one of the two disordered B(Mes)_2_ groups is shown. The central [K(18-crown-6)]^+^ moiety is disordered by 50% *via* inversion symmetry, with the K atom shifted off-center towards the [5]-helicene molecules. Both centrosymmetric configurations, each occupied by 50%, are shown.

The structures of the monoanions **1·K1** and **2·K1** were confirmed by single-crystal X-ray diffraction, as depicted in Fig. 1. We were unable to obtain single crystals of the dianions **1·K2** and **2·K2**. However, a 1 : 1 co-crystal of **2·K1** and **2·K2** was formed (**2·K1**/**2·K2**, Fig. 1c). In **2·K1**/**2·K2**, the B(Mes)_2_ group is disordered and one of the K(18-crown-6) moieties lies on an inversion center and is completely disordered *via* inversion symmetry (see Fig. 2). The potassium atom is offset from the inversion center by 50% to the ‘outer’ helicene and 50% to the centrosymmetric one. Hence, 50% dianion and 50% monoanion are present in the crystal structure and are disordered *via* inversion. We assume that both mono- and dianion were present and co-crystallized resulting in 1.5 K(18-crown-6) moieties per molecule. It is most likely that the monoanion and dianion have very similar molecular configurations and that the influence of the second reduction on the structural parameters is relatively small. However, the presence of slightly different geometries is reflected in enlarged anisotropic displacement parameters and in the disorder of the B(Mes)_2_ moiety; thus, it is not possible to comment further on bond distances within the monoanionic and dianionic helicene units.

In **1·K1**, no interaction is observed between the potassium cation and the helicene core. Instead, the potassium ion is coordinated by 18-crown-6 and two THF molecules (Fig. 1a). However, in **2·K1** and the co-crystal **2·K1**/**2·K2**, the potassium ions interact weakly with the helicene moieties, *i.e.*, with the helical terminal ring P1 bonded to boron and the helical ring P7 next to P1 (Fig. 1b, c and S4 in the ESI‡). While the anions and cations stack in an alternating fashion in **2·K1** (Fig. S4‡), in **2·K1**/**2·K2** the mono- and dianion alternate with the cations exhibiting a disordered, centrosymmetric sandwich packing arrangement with a THF–K(18-crown-6)–helicene–K(18-crown-6)–helicene–K(18-crown-6)–THF sequence (Fig. 2). The K⋯C distances are in the range 3.206(3)–3.534(3) Å in **2·K1**, and 3.083(4)–3.461(4) Å in **2·K1**/**2·K2** (Table S2‡), respectively, which is considerably below the sum of the van der Waals radii of 4.45 Å. Notably, there is no direct interaction between the potassium ion and the nitrogen or boron atoms. However, in the singly reduced species, **1·K1**, **2·K1** and **2·K1**/**2·K2**, the B–C(helicene) bond is significantly shortened by −0.024 to −0.035 (3) Å with respect to their neutral state, and possibly further for **2·K1**/**2·K2**, but the esds for the latter structure do not allow an accurate analysis (Table S2‡), which can be explained by the enhancement of π–B–C(helicene) character *via* the newly populated MOs (*vide infra*). As a result, the B–C(mesitylene) bonds are slightly lengthened by *ca.* +0.012 to +0.026 (2–4) Å in the radical monoanions. In the monoanion **1·K1**, the torsion angle between the BC_3_ plane and the helical terminal ring P1 (4.93(7)°) is significantly smaller than that in the neutral compound **1** (24.30(6)°). In the reduced anions **2·K1** and **2·K1**/**2·K2**, torsion angles are similar to those in the neutral species **2**. However, as noted above, interaction is observed between the potassium ion and the helicene core next to boron (P1 and P7). These results indicate delocalization of the single electron between boron and the helicene core. With respect to the NC_3_ plane, a decrease of the sum of C–N–C angles is observed from 359.40(14)° and 359.98(11)° in the neutral compounds **1** and **2**, respectively, to 355.51(16)° and 355.5(2)° in the reduced compounds (Table S2‡), which indicates some degree of localization of the N lone pair at the nitrogen atom in the latter.

**Fig. 3:**
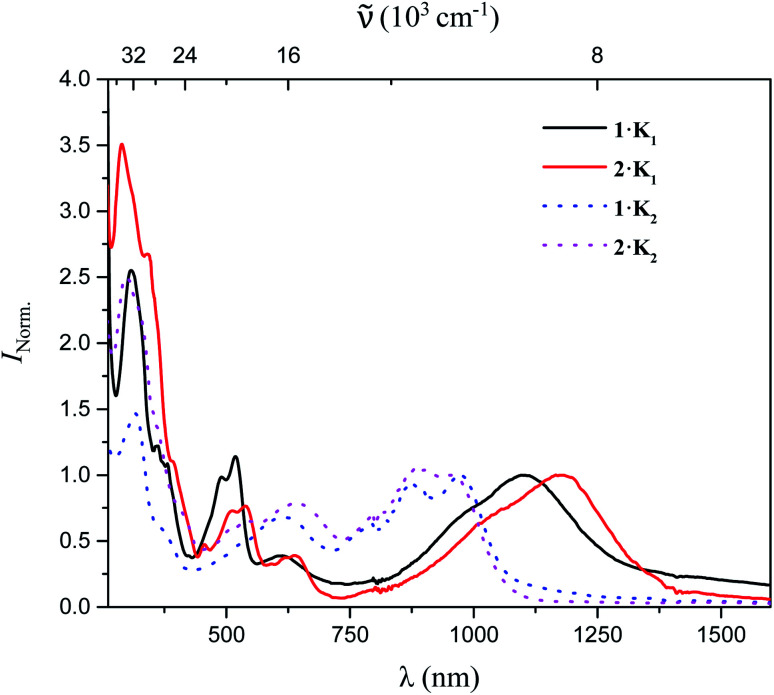
UV/vis-NIR absorption spectra of **1·K1**, **2·K1**, **1·K2**, and **2·K2** in Et_2_O.

The UV/vis-NIR absorption spectra of **1·K1**, **2·K1**, **1·K2**, and **2·K2** were recorded in Et_2_O under argon (Fig. 3). The lowest-energy bands of **1·K1**, **2·K1**, **1·K2**, and **2·K2** appear at 1104, 1176, 972 and 954 nm, respectively, which are dramatically red-shifted compared with those of the neutral compounds **1** and **2** (398 and 433 nm for **1** and **2** in THF, respectively). In contrast to the intense emission from solutions of neutral compounds **1** and **2**,^8^ there was no detectable emission from the monoanions and dianions in the NIR region.

**Fig. 4:**
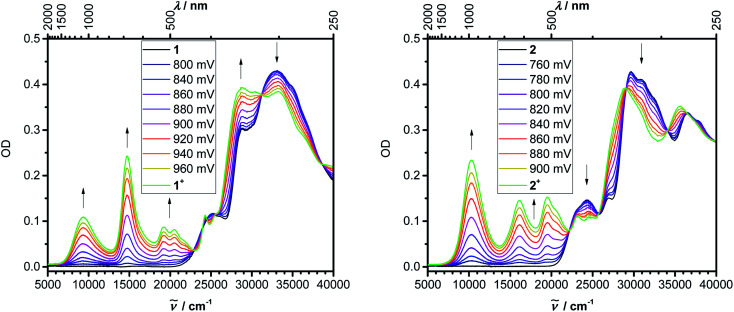
UV/vis-NIR spectroelectrochemistry of the oxidation of **1** and **2** in DCM (0.1 M [*n*Bu_4_N][PF_6_]).

Even though we were not successful in isolating the products of the chemical oxidation of compounds **1** and **2**, which is in agreement with the observation that some triarylamines tend to form unstable short-lived radicals,^12^ we were able to investigate the properties of the radical cations by performing UV/vis-NIR spectroelectrochemical measurements in CH_2_Cl_2_/0.1 M [*n*Bu_4_N][PF_6_] as depicted in Fig. 4. The lowest-energy absorptions of cations of **1** and **2** occur at 1071 and 972 nm, respectively.

**Fig. 5:**
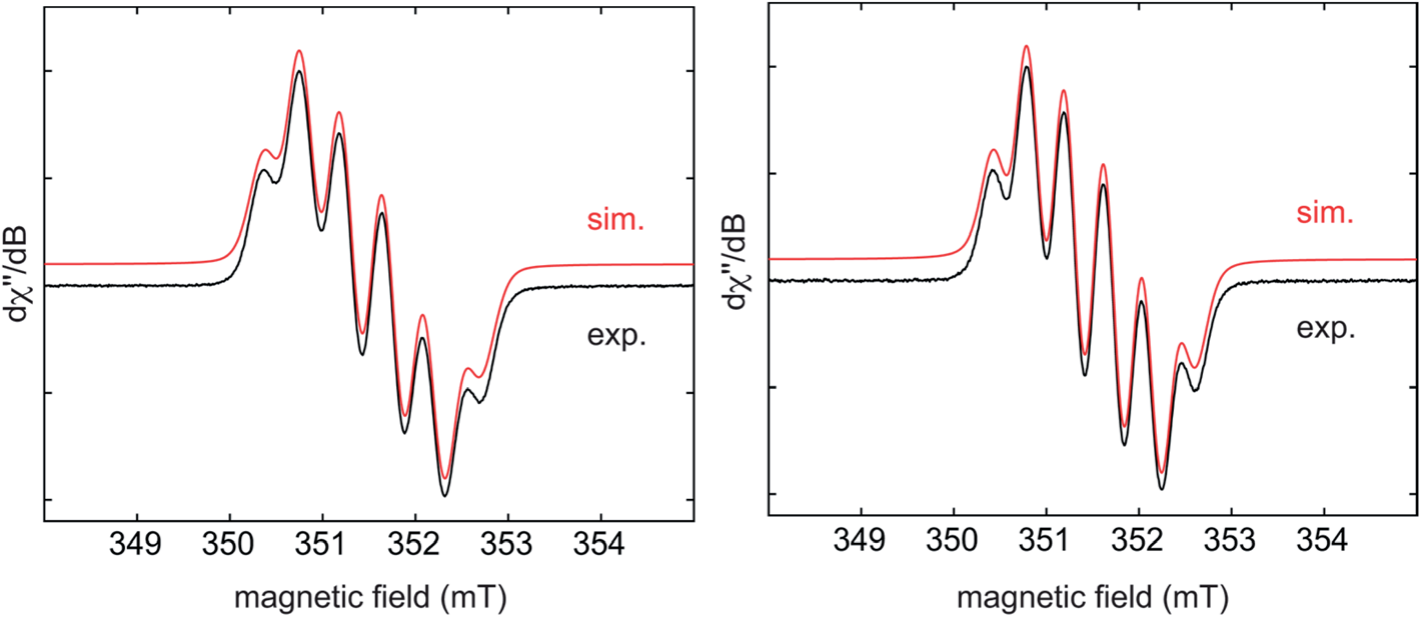
Experimental (black) and simulated (red) CW X-band EPR spectra of **1·K1** (left) and **2·K1** (right) in THF at room temperature. The simulation parameters are as follows: for **1·K1**, *g*_iso_ = 2.0028, *a*(B) = 12.7 MHz (4.53 G), *a*(H) = 10.8 MHz (3.85 G), 9.2 MHz (3.28 G), and 1.7 MHz (0.61 G); for **2·K1**, *g*_iso_ = 2.0028, *a*(B) = 11.9 MHz (4.25 G), *a*(H) = 10.0 MHz (3.57 G), 8.8 MHz (3.14 G), and 2.3 MHz (0.82 G).

EPR spectra of the radical monoanions of **1·K1** and **2·K1** were measured in THF at room temperature (Fig. 5). These species have boron hyperfine couplings (*g*_iso_ = 2.003, *a*(^11^B) = 4.5 G for **1·K1** and *g*_iso_ = 2.003, *a*(^11^B) = 4.2 G for **2·K1**, respectively) which are smaller than those of other triarylborane radical monoanions, such as [Ph_3_B]^−^ (7.84 G)^5*d*^ and [Mes_3_B]^−^ (9.87 G).^13^ The relatively low values for the boron hyperfine couplings indicate that the spin density is delocalized onto the annulated rings of the respective helicenes in **1·K1** and **2·K1**, which is in agreement with the calculated spin densities of these monoanions, *vide infra*. A good fit to the experimental data is obtained by considering couplings to the three protons of the adjacent benzene ring of the helicene.

**Fig. 6:**
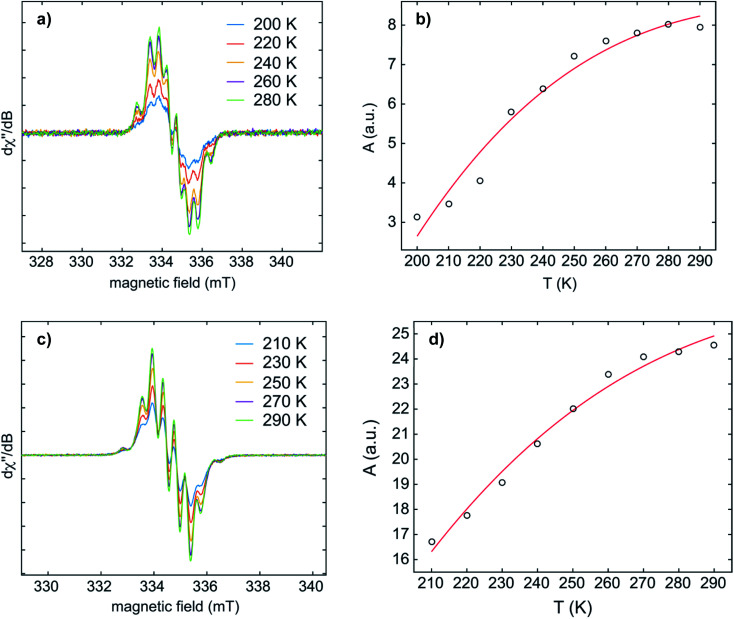
The dependence of the CW X-band EPR spectra (left) and double-integrated EPR intensity (A) (right) on the temperature of **1·K2** (a and b) and **2·K2** (c and d) in THF. Bleaney–Bowers fits to the variable-temperature EPR data gave S–T energy gaps of 4.3 and 4.7 kJ mol^−1^ for **1·K2** and **2·K2**, respectively.

Temperature-dependent EPR spectra of **1·K2** and **2·K2** were recorded in THF (Fig. 6 and S5–S8‡). Fitting the Bleaney–Bowers equation to our data provided the singlet–triplet (S–T) energy gaps of 4.3 and 4.7 kJ mol^−1^ for **1·K2** and **2·K2**, respectively. This small energy difference leads to an equilibrium between the two states at room temperature, where the S and T population ratios are 85 : 15 and 87 : 13 for **1·K2** and **2·K2**, respectively. It is important to note that we cannot exclude the presence of small amounts of the monoanions in the samples of **1·K2** and **2·K2**. Solid state EPR spectra of **1·K2** and **2·K2** were recorded at 145 K (Fig. S9 and S10‡). The weak half-field signal at 167.5 mT observed for **1·K2** is characteristic of the triplet state. However, for **2·K2**, the corresponding half-field signal could not be observed.

**Fig. 7:**
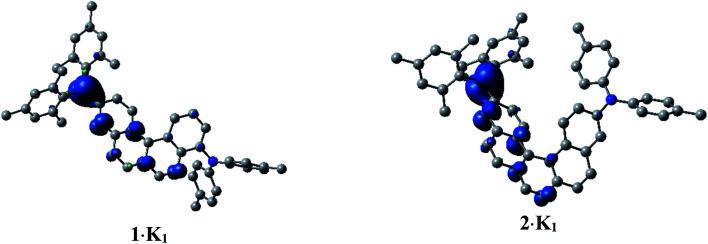
Spin density plots of **1·K1** (doublet) and **2·K1** (doublet), calculated as naked **11−** and **21−** species. H-atoms are omitted for clarity. Isovalue: ± 0.003 [*ea*_0_^−3^]^1/2^.

In order to investigate the electronic structure of **1·K1** and **2·K1**, we performed spin-unrestricted DFT calculations on their corresponding naked radical monoanions **11−** and **21−** featuring doublet ground states. The geometries were optimized without symmetry constraints using the UM062X functional^14^ in combination with the 6-31+G(d) basis set.^15^ Spin density plots (Fig. 7) of **11−** and **21−** indicate that the radicals are delocalized over the annulated helicene rings and the boron p_*z*_ orbital. This agrees well with the EPR measurements. The simulated UV/vis-NIR absorption spectrum of **11−** displays two low energy local excited (LE) transitions at 1117 and 917 nm (Table S3‡), respectively, in excellent agreement with the experimentally observed transitions at 1104 and 990 nm. These transitions have relatively high oscillator strengths and are mostly transitions between the SOMO and LUMO (Fig. S13‡), which lead to LE states. The lowest energy transitions for the radical **21−** were calculated to be at 1187 and 977 nm (exp.: 1176 and 1030 nm). While the major contribution to the first excited state is again attributed to a SOMO → LUMO transition, the second excited state involves contributions from LUMO+1 to LUMO+4, with both states characterized as LE states.

A multireference approach such as NEVPT2 is able to describe open-shell biradical structures very accurately. However, it is too demanding for the structures **1·K2** and **2·K2** without using model systems and, in addition, some of us found that the single-reference broken-symmetry DFT approaches are often sufficiently accurate.^16^ Thus, calculations for dianions **1·K2** and **2·K2** (as the naked species **12−** and **22−**) were carried out using the UM062X functional and the 6-31++G(d) basis set in combination with the SMD variation of PCM.^17^ The HOMO and LUMO in the unrestricted (UM062X) method were allowed to mix in order to destroy α–β and spatial symmetries. Within this approximation, an open-shell biradical S_0_ state would be described as a mixture of the singlet and triplet states, as indicated by an expectation value (〈*S*^2^〉) of about 1.0. If the energy difference between the orbitals is sufficiently small but not zero, the 〈*S*^2^〉 value is between one and zero, and the system can be described as “biradicaloid”.^18^ The energy splitting arises from possible interactions between both radical centers. For the dianions **1·K2** and **2·K2**, biradicaloid S_0_ open-shell systems are found to be the most stable electron configurations (Table 1). The small 〈*S*^2^〉 values of 0.3188 and 0.3950 indicate significant admixture of the S_0_ closed-shell state to the open-shell wavefunction. However, we recognize a strong influence of the environmental model, *e.g.*, gas phase calculations give larger values (〈*S*^2^〉 = 0.8097 for **1·K2** and 〈*S*^2^〉 = 0.8048 for **2·K2**), but the relative stability of open-shell S_0_ remains the same (Table S6‡). The calculated adiabatic singlet-triplet energy gaps are 16.4 kJ mol^−1^ for the dianion **1·K2** and 11.6 kJ mol^−1^ for **2·K2**, which is in a reasonable agreement with the Bleaney–Bowers fits of the variable-temperature EPR data. Frontier molecular orbitals (FMOs) of **1·K2** and **2·K2** as open-shell singlet and triplet with and without SMD are shown in Fig. S14–S21.‡ There is significant B(p_*z*_)–aryl(helicene) interaction in the FMOs of the open-shell singlet structures, which leads to a shortening of the B–C bond lengths of −0.013 to −0.018 Å with respect to their neutral states (Tables S4 and S5‡), and which is in agreement with the experimentally obtained X-ray data for **2·K1**/**2·K2** (*vide supra*). The energy difference between the two highest α and β orbitals (Δ*E*_αβ_) is 31.36 kJ mol^−1^ for **1·K2** and 23.35 kJ mol^−1^ for **2·K2**, respectively. However, larger splittings are found for the gas phase calculation (Δ*E*_αβ_ = 39.85 and 37.92 kJ mol^−1^ for **1·K2** and **2·K2**), in which we observe significant demixing between open-shell and closed-shell singlet states as indicated by the larger 〈*S*^2^〉 values in the gas phase.

**Table 1:** Expectation values and relative energies of lowest states for the dianions **1·K2** and **2·K2** (as **12−** and **22−**). Calculations were performed at the (U)M062X/6-31++G(d)/SMD = Et_2_O level of theory

	State	〈*S*^2^〉	Δ*E*^a^ (kJ mol^−1^)	Δ*E*_αβ_^b^ (kJ mol^−1^)
**12−**	S_0_ (open-shell)	0.3188	0	+31
	S_0_ (closed-shell)	0.0000	+2	—
	T_1_ (vertical)^c^	2.0193	+47	—
	T_1_	2.0261	+16	—
**22−**	S_0_ (open-shell)	0.3950	0	+23
	S_0_ (closed-shell)	0.0000	+34	—
	T_1_ (vertical)^c^	2.0255	+39	—
	T_1_	2.0355	+12	—

a Energy relative to S_0_ open-shell system.

b Energy difference between highest α–β in the S_0_ open-shell system.

c Geometry of S_0_ open-shell.

In general, the energy difference between closed- and open-shell singlet and triplet states in these systems is small, which is in qualitative agreement with the EPR spectra (*vide supra*). The overestimation of the singlet-triplet energy gap might result because the influence of the counter-ion is neglected.^19^ This was probed by a single point calculation on the experimental geometrical parameters obtained for **2·K1**/**2·K2**, *i.e.*, in the presence of the counterions. While 〈*S*^2^〉 drops to 0.1537 indicating significant demixing, the vertical singlet triplet gap decreases to 18 kJ mol^−1^. Thus, it is feasible that the adiabatic gap is also further decreased. Additionally, due to the delocalization error of DFT functionals,^20^ the interaction between both radical centers might be overestimated. Computations to investigate the influence of both error sources are under way.

## Conclusions

We have reduced triarylborane-based helical donor–acceptor compounds **1** and **2** stepwise and obtained the corresponding monoanions and dianions. We were able to isolate and structurally characterize the monoanions **1·K1**, **2·K1** and a 1 : 1 co-crystal of **2·K1**/**2·K2**. All species (**1·K1**, **2·K1**, **1·K2** and **2·K2**) were investigated by UV/vis-NIR absorption, EPR spectroscopy, and theoretical calculations. DFT calculations and the EPR analysis clearly revealed that the unpaired electron in both radical anions is delocalized over the annulated helicene rings and the boron p_*z*_ orbital. This is supported by changes observed in the crystal structures on reduction, *e.g.*, the B–C(helicene) bond shortening. The broken-symmetry DFT approach indicates that both dianions **1·K2** and **2·K2** can best be described as biradicaloid systems with significant admixing of the closed-shell S_0_ state. In addition, the properties of the radical cations of **1** and **2** were investigated by spectroelectrochemistry. Our results provide insights into helicene-based redox-active systems which may prove useful in the design of new helicenes for applications in materials chemistry.

## Data availability

All experimental procedures, computational data, *xyz* coordinates for the calculated structures, and spectroscopic data can be found in the ESI.‡

## Author contributions

X. J. and Z. W. performed the experiments; J. N. and F. F. did the DFT calculations supervised by B. E.; A. F., Z. W. and J. K. carried out X-ray crystallographic studies; I. K. carried out EPR spectroscopy supervised by H. B.; M. M. carried out spectroelectrochemical measurements supervised by C. L.; T. B. M. supervised the overall project; all authors were involved in the preparation of the manuscript.

## Conflicts of interest

The authors declare no conflict of interest.

## Supplementary Material

SC-012-D1SC02409D-s001

SC-012-D1SC02409D-s002
