# Supplementary material for: One- and two-electron reduction of triarylborane-based helical donor–acceptor compounds
Source: Chem Sci. 2021 Jul 27;12(35):11864–72. doi: 10.1039/d1sc02409d (PMC8442707; doi:10.1039/d1sc02409d)
Supplement: SC-012-D1SC02409D-s001 [file SC-012-D1SC02409D-s001.pdf]

Electronic Supplementary Material (ESI) for Chemical Science.
This journal is © The Royal Society of Chemistry 2021

Electronic Supporting Information

One- and Two-Electron Reduction of Triarylborane-Based Helical Donor-Acceptor Compounds

Xiangqing Jia,[a] Jörn Nitsch,[a] Zhu Wu,[a] Alexandra Friedrich,[a] Johannes Krebs,[a] Ivo Krummenacher,[a] Felipe Fantuzzi,[a,c] Holger Braunschweig,[a] Michael Moos,[b] Christoph Lambert,[b] Bernd Engels[c] and Todd B. Marder*[a]

[a] Institut für Anorganische Chemie and Institute for Sustainable Chemistry & Catalysis with Boron, Julius-Maximilians-Universität Würzburg, Am Hubland, 97074 Würzburg, Germany

[b] Institut für Organische Chemie, Julius-Maximilians-Universität Würzburg, Am Hubland, 97074 Würzburg, Germany

[c] Institut für Physikalische and Theoretische Chemie, Julius-Maximilians-Universität Würzburg, Am Hubland, 97074 Würzburg, Germany

*Email: todd.marder@uni-wuerzburg.de

Table of contents……………………………………………………….......S1

General information……………………………………………………………..S2

Synthesis of monoanions and dianions of compounds **1** and **2**…………...…………..S2

Single-crystal X-ray diffraction……………………………………………………S5

EPR measurements………………………………………………………………S9

Photophysical properties………………………………………………………...S11

Spectroelectrochemical measurements……………………………….……S11

Theoretical studies…………………………………………………….……...S12

References…………………………………………………………………...….S25

XYZ-coordinates……………………………………………………….……..S25

General information

Compounds **1**[1], **2**[1] and [K(18-crown-6)(THF)$_2$] naphthalenide[2] were synthesized according to literature procedures. THF and Et_2O were dried using an Innovative Technology Inc. solvent purification system (SPS), pentane was distilled from $LiAlH_4$, and all were stored over Na/K alloy in an argon-filled glovebox. Electronic absorption measurements were performed on a Varian Cary 5E UV/vis-NIR spectrophotometer and an Agilent 8453 diode-array UV/vis spectrophotometer. ^{1}H NMR spectra were recorded on a Bruker Avance 500 MHz (^{1}H, 500 MHz) spectrometer at room temperature. The residual peaks of the deuterated solvents were used as references for ^{1}H chemical shifts.

Synthesis of monoanions and dianions of compounds 1 and 2

*Synthesis of **1·K₁***

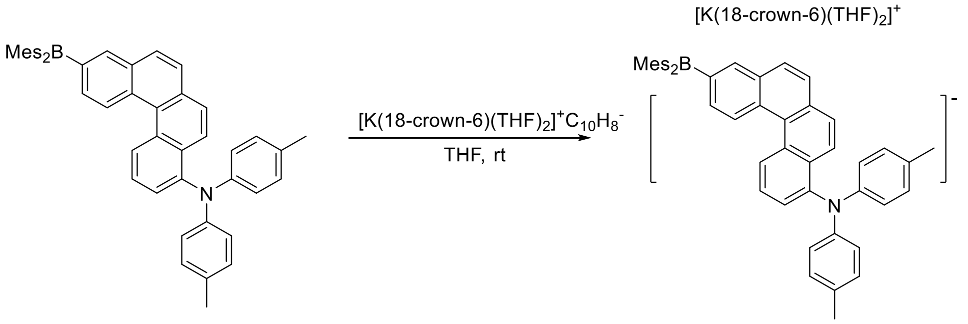

In an argon-filled glovebox, **1** (10 mg, 0.0149 mmol, 1.0 equiv.), [K(18-crown-6)(THF)$_2$]$^+$ naphthenide (9.4 mg, 0.0164 mmol, 1.1 equiv.), and THF (1 mL) were added into a 5 mL vial, and the mixture was stirred for 5 min to form a dark purple solution. The lid of the vial was removed, and the vial was placed inside a 25 mL vial containing pentane. The large vial was sealed and placed in a freezer in the glovebox (-30 °C). After one week, dark purple crystals formed and the structure was confirmed by single-crystal X-ray diffraction.

*Synthesis of **1·K₂***

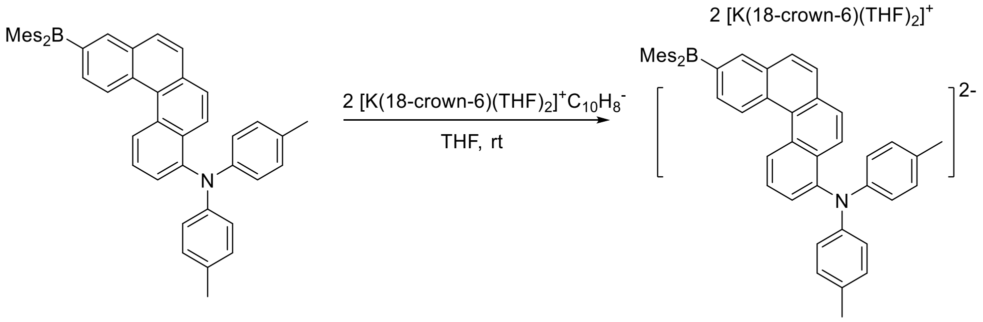

In an argon-filled glovebox, **1** (10 mg, 0.0149 mmol, 1 equiv.), [K(18-crown-6)(THF)$_2$]$^+$ naphthenide (21.4 mg, 0.0372 mmol, 2.5 equiv.), and THF (1 mL) were added into a 5 mL vial, and the mixture was stirred for 10 min to form a dark blue solution. The lid of the vial was removed, and the vial was placed inside a 25 mL vial containing pentane. The large vial was sealed and placed in a freezer in the glovebox (-30 °C). After 3 days, dark blue powder precipitated. Because of biradicaloid character, the ^{11}B and ^{13}C NMR were not obtained. ^{1}H NMR (500 MHz, THF-d$_8$): δ = 6.88-6.81 (m, 8H), 6.37 (s, 2H), 6.36 (s, 2H), 5.45 (d, *J* = 7.5 Hz, 1H), 5.21 (d, *J* = 7.5 Hz, 1H), 4.89 (d, *J* = 9.5 Hz, 1H), 4.84 (t, *J* = 7.5 Hz, 1H), 4.40 (d, *J* = 9.5 Hz, 1H), 3.71 (s, 2H), 3.55 (s, 1H), 3.47 (d, *J* = 7.0 Hz, 1H), 3.27 (d, *J* = 7.0 Hz, 1H), 2.18 (s, 6H), 2.10-1.98 (m, 18H) ppm.

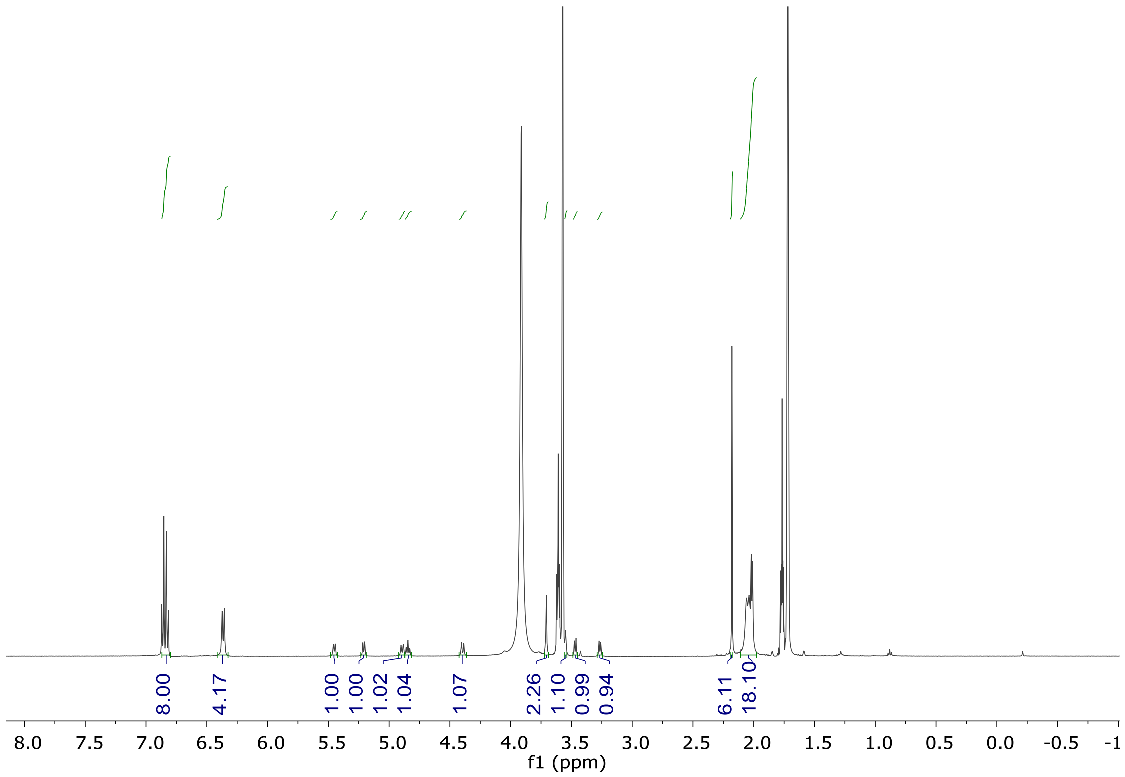

Figure S1. ^{1}H NMR spectrum of **1·K$_2$** in THF-d$_8$ at 500 MHz

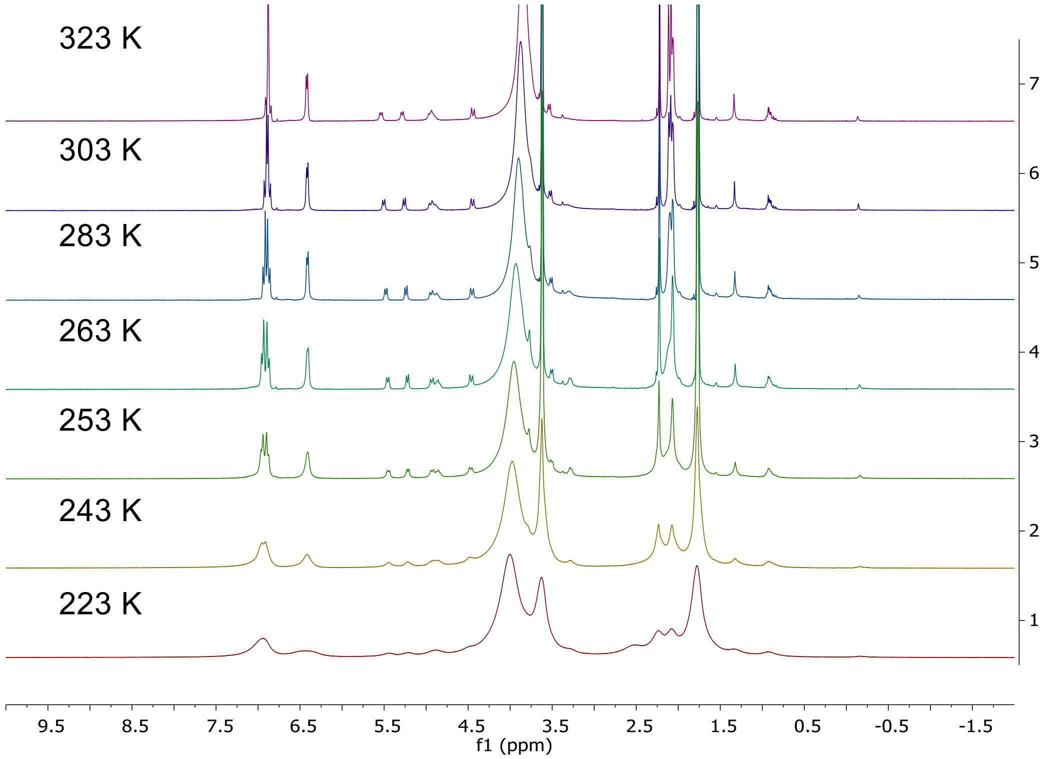

Figure S2. Temperature-dependent ^{1}H NMR spectra of **1·K$_2$** in THF-d$_8$ at 500 MHz

*Synthesis of **2·K₁***

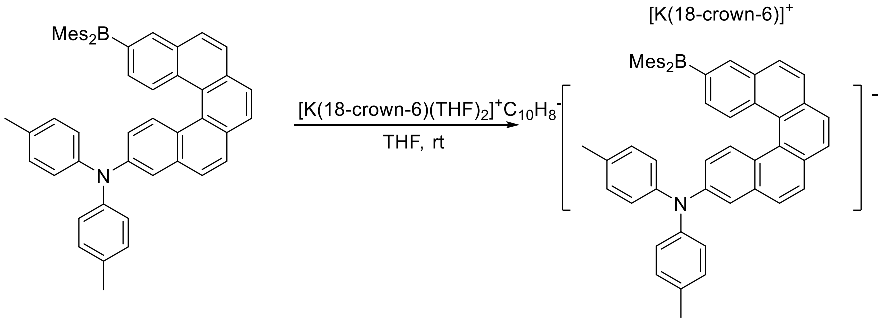

In an argon-filled glovebox, **2** (10 mg, 0.0139 mmol, 1 equiv.), $[K(18\text{-crown-}6)(THF)_2]^+$ naphthenide (8.7 mg, 0.0152 mmol, 1.1 equiv.), and THF (1 mL) were added into a 5 mL vial, and the mixture was stirred for 5 min to form a dark purple solution. The lid of the vial was removed, and the vial was placed inside a 25 mL vial containing pentane. The large vial was sealed and placed in a freezer in the glovebox (-30 °C). After one week, dark purple crystals formed and the structure was confirmed by single-crystal X-ray diffraction.

*Synthesis of **2·K₂***

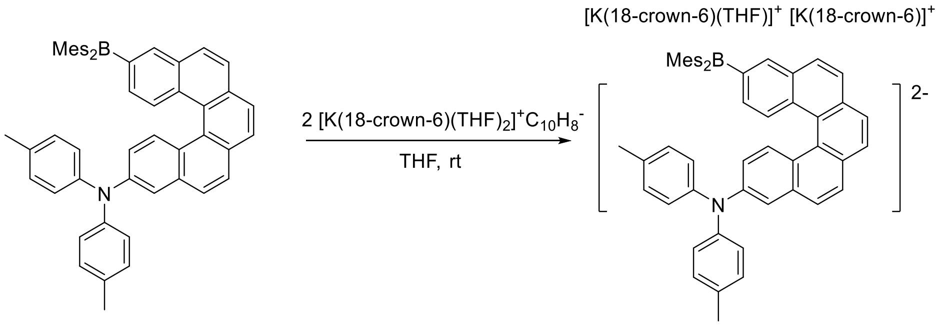

In an argon-filled glovebox, **2** (10 mg, 0.0139 mmol, 1 equiv.), $[K(18\text{-crown-}6)(THF)_2]^+$ naphthenide (20 mg, 0.0346 mmol, 2.5 equiv.), and THF (1 mL) were added into a 5 mL vial, and the mixture was stirred for 10 min to form a dark blue solution. The lid of the vial was removed, and the vial was placed inside a 25 mL vial containing pentane. The large vial was sealed and placed in a freezer in the glovebox (-30 °C). After one week, dark blue crystals formed. Single crystal X-ray diffraction analysis revealed a disordered 1:1 co-crystal of the monoanion **$2{\cdot}K_1$** and the dianion **$2{\cdot}K_2$**. Because of biradicaloid character and trace of **$2{\cdot}K_1$** in it, the ^{11}B and ^{13}C NMR were not obtained. ^1H NMR (500 MHz, THF-d_8): δ = 6.97, 6.62, 6.57, 6.33, 6.25, 5.47, 5.19, 4.82, 4.62, 4.48, 4.19, 4.13, 3.47, 3.38, 2.55, 2.35, 2.31, 2.15, 2.09, 1.83, 1.72 ppm.

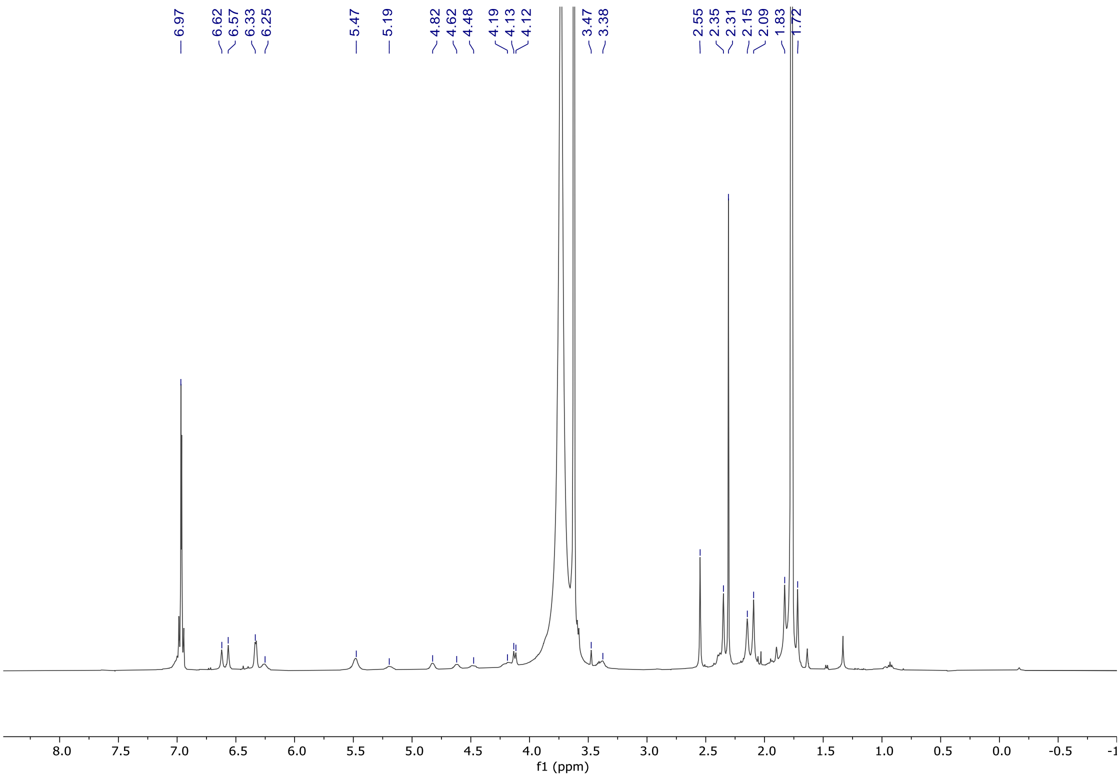

Figure S3. ^{1}H NMR spectrum of **2·K$_2$** in THF-d$_8$ at 500 MHz

Single-crystal X-ray diffraction

Crystals suitable for single-crystal X-ray diffraction were selected, coated in perfluoropolyether oil, and mounted on MiTeGen sample holders. Diffraction data for **1·K$_1$** and 1:1 **2·K$_1$ / 2·K$_2$** were collected on a Rigaku Oxford Diffraction XtaLAB Synergy diffractometer with a semiconductor HPA-detector (HyPix-6000) and multi-layer mirror monochromated Cu-K$_\alpha$ radiation. Diffraction data for **2·K$_1$** were collected on a Bruker X8 Apex II 4-circle diffractometer with a CCD area detector using Mo-K$_\alpha$ radiation generated by a Nonius FR591 rotating anode and monochromated by multi-layer focusing mirrors. As these crystals were extremely unstable in air, they were rapidly mounted under an argon stream and cooled at 100 K using an Oxford Cryostream low-temperature device attached to the diffractometer. Data were collected at 100 K. The images were processed and corrected for Lorentz-polarization effects and absorption as implemented in the CrysAlis^{Pro} software from Rigaku Oxford Diffraction (**1·K$_1$** and 1:1 **2·K$_1$ / 2·K$_2$**) or in the Bruker software packages (**2·K$_1$**). The structures were solved using the intrinsic phasing method (SHELXT)[3] and Fourier expansion technique. All non-hydrogen atoms were refined in anisotropic approximation, with hydrogen atoms "riding" in idealized positions, by full-matrix least squares against F^2 of all data, using SHELXL software[4] and the SHELXLE graphical user interface.[5] The crystal of **1·K$_1$** consisted of two domains and, hence, a twin data reduction was performed. Only the larger domain (ca. 80%) was used in the structure refinement. Mercury[6] and Diamond software[7] were used for graphical representation. Crystal data and experimental details are listed in Table S1. CCDC 2044266 (**1·K$_1$**), 2044267 (**2·K$_1$**), and 2044268 (**2·K$_1$ / 2·K$_2$**) contain the supplementary crystallographic data for this paper. These data are provided free of charge by The Cambridge Crystallographic Data Centre.

Table S1. Single-crystal X-ray diffraction data and structure refinements of **1·K$_1$**, **2·K$_1$** and the 1:1 co-crystal of **2·K$_1$ / 2·K$_2$**.

	**1·K$_1$**	**2·K$_1$**	**2·K$_1$ / 2·K$_2$**
CCDC	2044266	2044267	2044268
Empirical formula	$C_{50}H_{46}BN \cdot C_{12}H_{24}KO_6 \cdot 3(C_4H_8O)$	$C_{54}H_{48}BN \cdot C_{12}H_{24}KO_6$	$C_{54}H_{48}BN \cdot 1.5(C_{12}H_{24}KO_6) \cdot C_4H_8O$
Formula weight (g·mol^{-1})	1191.41	1025.15	1248.96
Temperature (K)	100(2)	100(2)	100(2)
Radiation, λ (Å)	$Cu_{K\alpha}$ 1.54184	$Mo_{K\alpha}$ 0.71073	$Cu_{K\alpha}$ 1.54184
Crystal size (mm$^{3)}$	0.25 × 0.18 × 0.06	0.06 × 0.09 × 0.22	0.02 × 0.08 × 0.19
Crystal color, habit	black block	red needle	dark purple plate
Crystal system	triclinic	triclinic	triclinic
Space group	$P\bar{1}$	$P\bar{1}$	$P\bar{1}$
Unit cell dimensions			
a (Å)	12.5786(3)	8.842(3)	13.5466(5)
b (Å)	16.3020(4)	13.762(6)	15.5380(6)
c (Å)	16.8804(3)	24.038(12)	18.5839(6)
α (°)	98.375(2)	73.438(9)	87.406(3)
β (°)	90.741(2)	86.260(12)	72.123(3)
γ (°)	103.958(2)	86.518(8)	67.804(3)
Volume (Å^3)	3319.24(13)	2795(2)	3435.8(2)
Z	2	2	2
ρ_{calc} (Mg·m^{-3})	1.192	1.218	1.207
μ (mm^{-1})	1.149	0.148	1.411
F(000)	1282.0	1094	1337
θ range / °	5.298 – 70.058	2.311 – 26.403	2.512 – 72.761
Reflections collected	42082	99934	124255
Unique reflections	12455	11400	12277
R_{int}	0.0583	0.1598	0.0901
R_σ	0.0339	0.1156	0.0359
Parameters / restraints	935 / 586	684 / 0	1119 / 793
GooF on F^2	1.056	0.996	1.056
R_1 [I≥2σ(I)]	0.060	0.0580	0.0917
wR^2 (all data)	0.1818	0.1311	0.2932
Max. / min. residual electron density (e·Å^{-3})	0.741 / -0.460	0.248 / -0.254	0.759 / -0.356

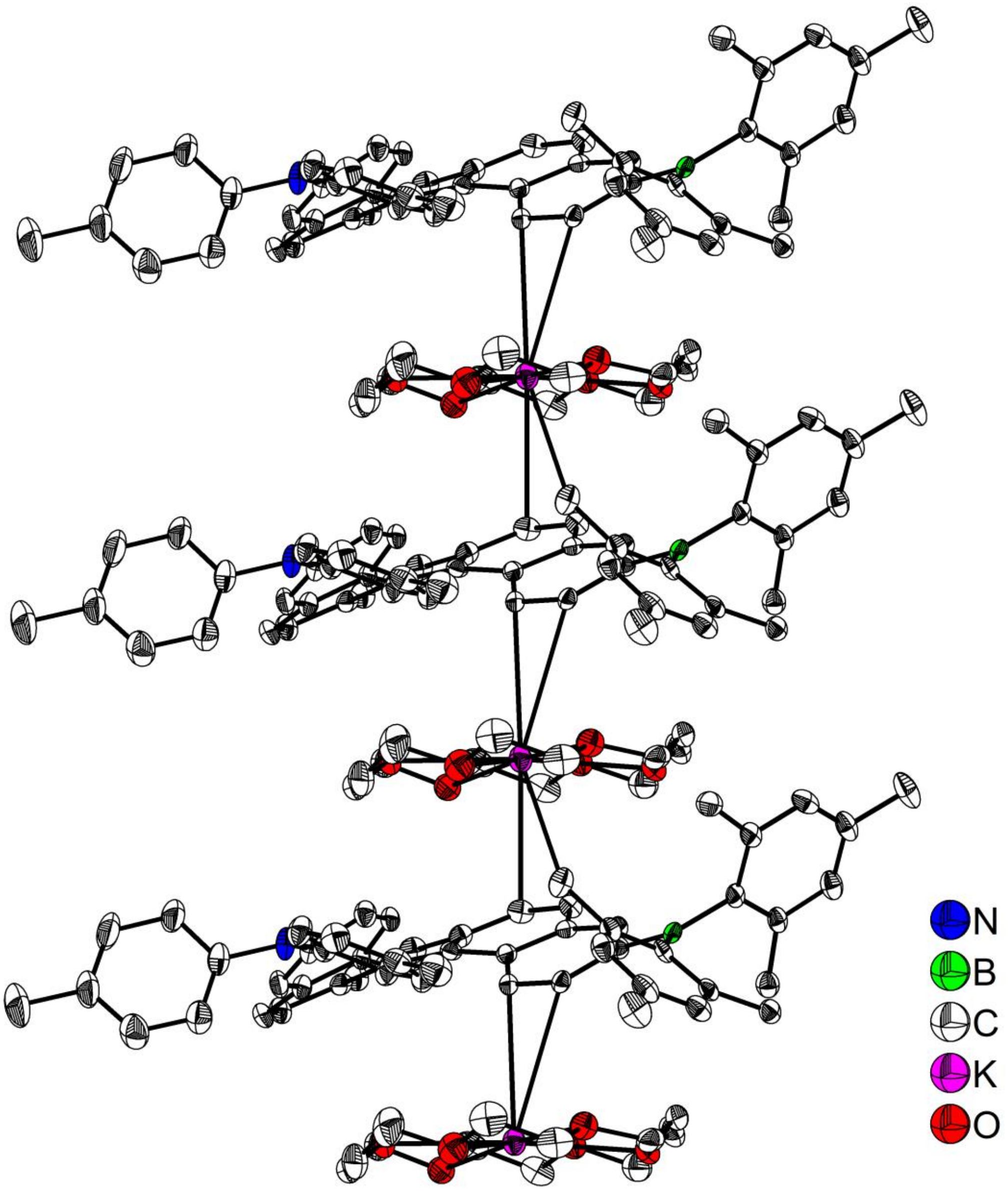

Figure S4. Alternate stacking arrangement of the [K(18-crown-6)]$^+$ cation and the [5]-helicene anion in **2·K$_1$** in the solid state at 100 K. Weak interactions exist between the potassium ion and the helicene core. Atomic displacement ellipsoids are drawn at 50% probability, and hydrogen atoms are omitted for clarity.

Table S2. Selected bond lengths [Å] and angles [°] for **1**, **1·K$_1$**, **2**, **2·K$_1$**, and **2·K$_1$ / 2·K$_2$** in the solid state at 100 K. Values for **1** and **2** are taken from our previous work.[1] P1 and P4 designate benzene rings of the helicene bonded to boron and nitrogen, respectively, while P2, P3 and P5, P6 designate phenyl rings of the mesityl groups bonded to boron and nitrogen, respectively. P7 is the benzene ring of the helicene next to P1.

Compound	**1**	**1·K$_1$**	**2**	**2·K$_1$**	**2·K$_1$ / 2·K$_2$** [a]
K···C19/C16 (P7)				3.283(3)	3.083(4)
K···C20/C15 (P7)				3.206(3)	3.461(4)
K···C22 (P3)				3.534(3)	3.249(5)
K···C21 (P3)				3.296(3)	3.326(4)
C–C (P7) [b]	1.354(2)	1.356(3)	1.351(3)	1.353(4)	1.362(6)
C–C (P3) [b]	1.366(2)	1.368(3)	1.367(3)	1.360(4)	1.356(6)
B–C_{Hel}	1.557(3)	1.533(3)	1.555(3)	1.520(4)	1.592(16) / 1.43(2)
B–C_{Mes}	1.575(3)	1.598(3)	1.585(2)	1.597(4)	1.579(16) / 1.63(2)
B–C_{Mes}	1.575(3)	1.601(3)	1.579(2)	1.605(4)	1.612(16) / 1.58(2)
N–C_{Hel}	1.428(2)	1.398(3)	1.411(2)	1.420(4)	1.437(5)
N–C_{Mes}	1.405(2)	1.434(3)	1.432(2)	1.436(4)	1.443(6)
N–C_{Mes}	1.430(2)	1.447(2)	1.412(2)	1.419(4)	1.405(6)
∠ $P1_{Hel}$-BC_3	24.30(6)	4.95(9)	12.80(5)	12.53(12)	10.3(2) / 5.3(3)
∠$P2_{Mes}$-BC_3	51.41(6)	60.86(8)	62.89(5)	57.19(11)	52.5(3) / 60.1(4)
∠ $P3_{Mes}$-BC_3	54.99(6)	63.78(8)	64.58(5)	60.28(11)	55.5(4) / 58.9(4)
∠ $P4_{Hel}$-NC_3	66.42(6)	77.18(8)	29.24(5)	32.19(13)	38.62(18)
∠ $P5_{Mes}$-NC_3	7.99(7)	14.90(9)	29.60(6)	27.38(13)	31.5(2)
∠ $P6_{Mes}$-NC_3	58.04(6)	57.82(9)	53.35(7)	65.44(12)	55.9(2)
∠ $P1_{Hel}$-$P4_{Hel}$	29.88(5)	32.04(7)	45.09(4)	39.74(10)	48.04(13)
∠ C–B–C	121.36(16)<br>119.65(16)<br>118.96(15)	120.48(16)<br>119.51(18)<br>119.97(18)	121.33(11)<br>118.21(12)<br>120.44(12)	120.4(2)<br>119.3(2)<br>120.2(2)	120.3(11) / 118.3(5)<br>122.8(10) / 115.5(13)<br>116.9(10) / 126.2(14)
∠ C–N–C	122.69(14)<br>121.76(14)<br>114.95(14)	121.91(16)<br>119.28(16)<br>114.32(16)	119.61(11)<br>122.63(11)<br>117.74(11)	117.2(2)<br>122.4(2)<br>115.9(2)	120.7(3)<br>119.9(3)<br>116.0(4)
Sum ∠ C–B–C	359.97(16)	359.96(18)	359.98(12)	359.9(2)	360.0(11) / 360.0(2)
Sum ∠ C–N–C	359.40(14)	355.51(16)	359.98(11)	355.5(2)	356.6(4)

[a] The $B(Mes)_2$ group is disordered. Hence, values are given for both parts.

[b] These are the respective C–C distances between the carbon atoms that exhibit interactions with the potassium ion in **2·K$_1$** and **2·K$_1$ / 2·K$_2$**.

EPR measurements

EPR measurements at X-band (9.85 GHz) were carried out at room temperature using a Bruker ELEXSYS E580 CW EPR spectrometer. The spectral simulations were performed using MATLAB 8.3 and the EasySpin 5.2.25 toolbox.[8] Temperature-dependent EPR measurements at X-band (9.4 GHz) were carried out using a Bruker ELEXSYS E580 CW EPR spectrometer equipped with an Oxford Instruments helium cryostat (ESR900) and a MercuryiTC temperature controller. Solid-state EPR measurements at X-band (9.38 GHz) were carried out using a Bruker ELEXSYS E580 CW EPR spectrometer.

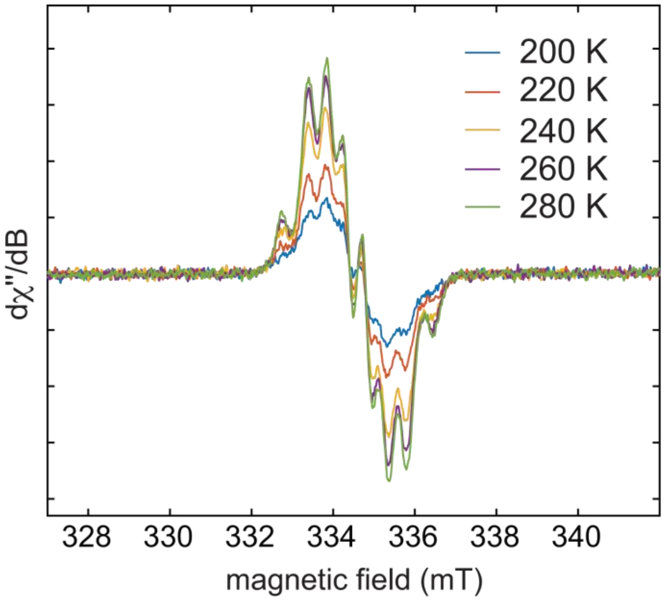

Figure S5. Temperature dependence of the CW X-band EPR spectra of **1·K$_2$** in frozen tetrahydrofuran.

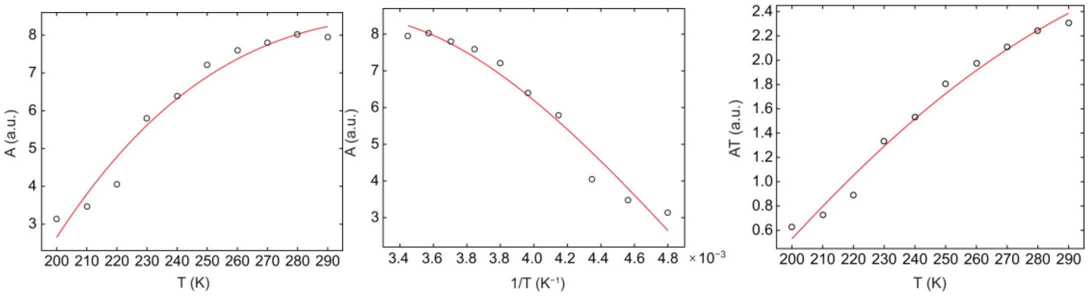

Figure S6. Three different representations of the temperature dependence of the double integral EPR intensity (A) of **1·K$_2$** in frozen solution. Circles (○) represent the experimental results and the red line corresponds to the fit with the Bleaney-Bowers equation. Analysis of the variable temperature EPR data gives a singlet-triplet gap of $2J = -360$ cm^{-1} ($\Delta E_{ST} = 4.3$ kJ/mol).

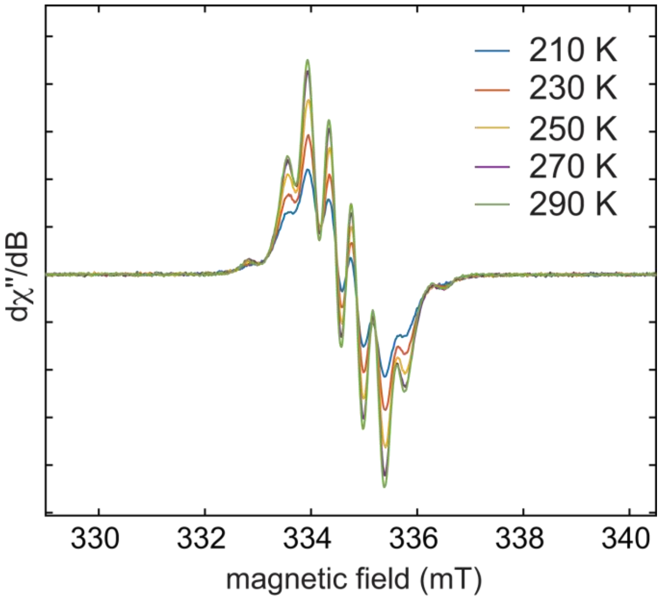

Figure S7. Temperature dependence of the X-band EPR spectra of **2·K$_2$** in frozen tetrahydrofuran.

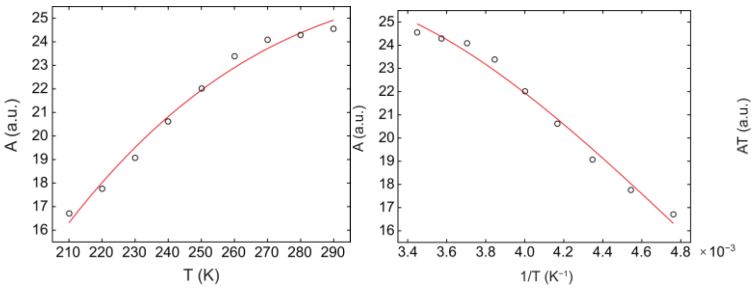

Figure S8. Three different representations of the temperature dependence of the double integral EPR intensity (A) of **2·K$_2$** in frozen solution. Circles (○) represent the experimental results and the red line corresponds to the best fit with the Bleaney-Bowers equation. Analysis of the variable temperature EPR data gives a singlet-triplet gap of $2J = -390$ cm^{-1} ($\Delta E_{ST} = 4.7$ kJ/mol).

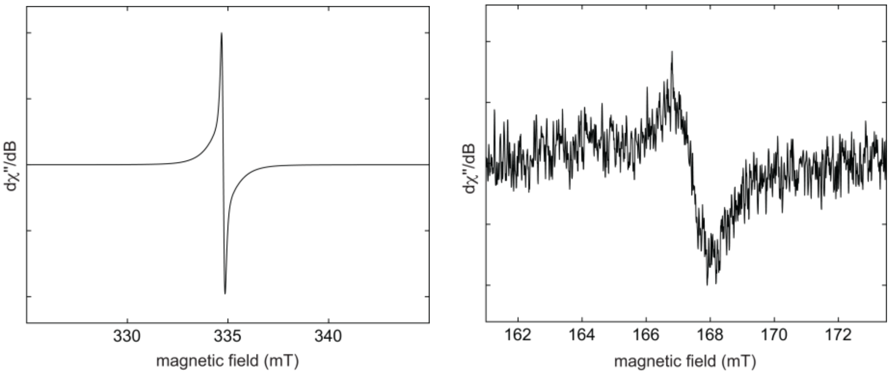

Figure S9. Solid-state CW X-band EPR spectrum of dianion **1·K$_2$** (*left*) at 145 K and the weak half-field signal at 167.5 mT observed for **1·K$_2$**, characteristic of the triplet state (*right*).

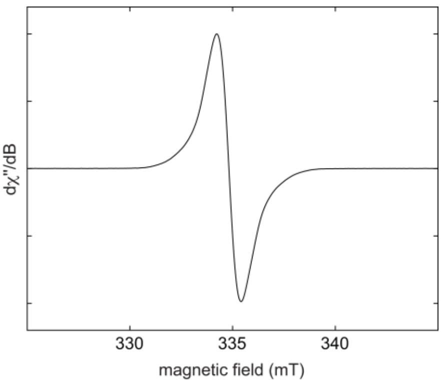

Figure S10. Solid-state CW X-band EPR spectrum of dianion **2·K$_2$** at 145 K. The corresponding half-field signal could not be observed at this temperature and also not in the temperature range of 120 to 300 K.

Photophysical properties

UV/vis-NIR absorption spectra were measured on a Varian Cary 5E UV/vis-NIR spectrophotometer and on an Agilent 8453 diode array UV/vis spectrophotometer. All solutions used in photophysical measurements had concentrations of ca. 10^{-5} M in Et_2O. All absorption spectra were recorded in standard quartz cuvettes (1 cm × 1 cm) under argon.

Spectroelectrochemical measurements

Spectroelectrochemical experiments in reflection mode were performed using an Agilent Cary 5000 spectrometer in combination with a custom designed sample compartment consisting of a cylindrical PTFE cell with an Infrasil® wedge window (angled by 0.5°) and an adjustable two-in-one electrode (6 mm platinum disc working electrode, 1 mm platinum wire counter electrode). The potentials were adjusted with a Princeton Applied Research potentiostat (PAR 283) and referenced to a leak free Ag/AgCl reference electrode (Warner Instruments). All experiments were carried out at room temperature under an argon atmosphere.

Thin layer measurements were done by attaching the working electrode to the flat surface of a glass halfsphere and measuring 9 cycles with a scan speed of 2 mVs^{-1}. The voltammograms were referenced to the ferrocene/ferrocenium redox couple.

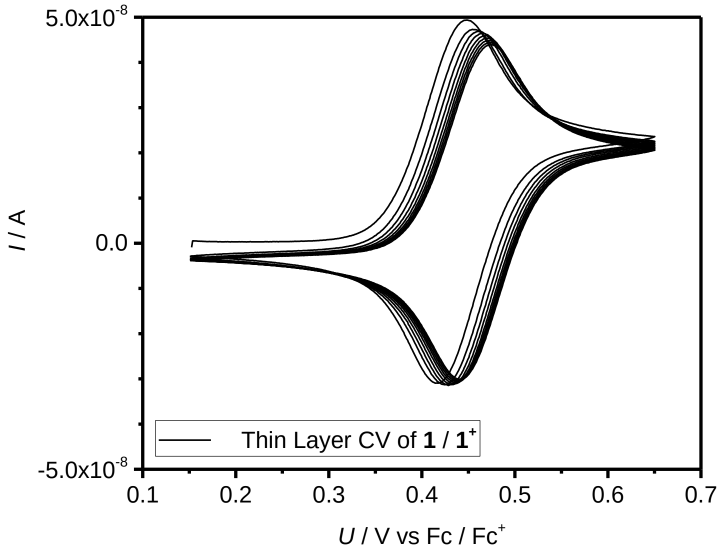

Figure S11. Thin layer cyclic voltammetry of compound **1** in dichloromethane (0.1 M [*n*Bu_4N][PF_6]).

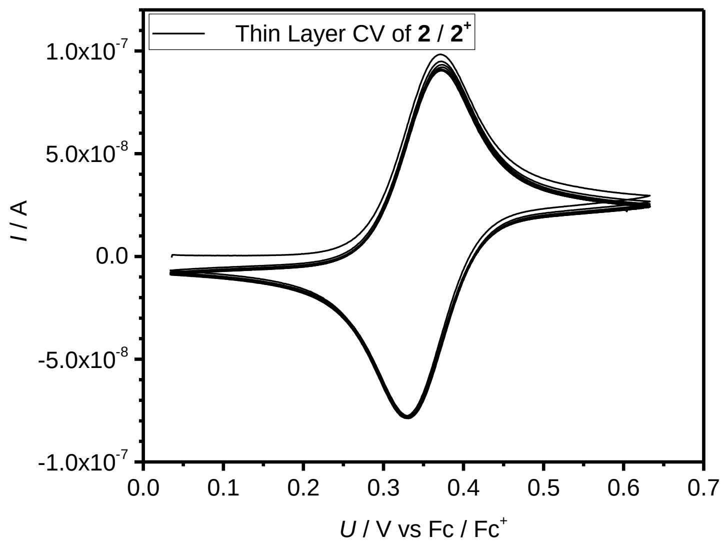

Figure S12. Thin layer cyclic voltammetry of compound **2** in dichloromethane (0.1 M [*n*Bu_4N][PF_6]).

Theoretical studies

DFT calculations were carried out with the program package Gaussian 16 (Rev. B.01).[9] The geometries were optimized without symmetry constraints using the (U)M062X functional[10] in combination with 6-31G+(d) and 6-31G++(d) basis set[11] supplemented by diffuse functions.[12] Calculations for dianions **1·K**$_2$ and **2·K**$_2$ were carried out using the (U)M062X functional and the 6-31G++(d) basis set in combination with Truhlar and co-workers' SMD variation of PCM.[13] The HOMOs and LUMOs in the unrestricted (UM062X) method were allowed to mix in order to destroy α-β and spatial symmetries. In a different approach, the stability of DFT

wavefunction was tested with stable=opt, which led to the same unrestricted wavefunctions. Gausview 6.0 was used to plot orbital surfaces. The lowest-energy vertical transitions were calculated by TD-DFT using the same level of theory and were further analyzed with the Multiwfn software.[14]

Table S3. Calculated lowest energy transitions for **1·K$_1$**, and **2·K$_1$**. LE = local excited state; D = doublet.

	State	Vertical Transition Energy[a]		Oscillator Strength *f*	MOs (%)[b]	Λ[c]	State Assignment
		[eV]	[nm]				
**1·K$_1$**	FC-D$_1$	1.11	1117 (1104)	0.1380	SOMO → LUMO (33)<br>SOMO → LUMO+1 (25)<br>SOMO → LUMO+2 (24)	0.568	LE
	FC-D$_2$	1.38	917 (990)	0.3194	SOMO → LUMO (54)	0.494	LE
**2·K$_1$**	FC-D$_1$	1.05	1184 (1176)	0.0071	SOMO → LUMO (65)<br>SOMO → LUMO+4 (17)	0.538	LE
	FC-D$_2$	1.27	977 (1030)	0.2932	SOMO → LUMO+1 (40)<br>SOMO → LUMO+2 (18)<br>SOMO → LUMO+4 (16)<br>SOMO → LUMO+3 (16)	0.599	LE

[a] Experimental values are given in brackets. [b] Major contributions are shown (>10%) [c] Tozer's Lambda index: *J. Chem. Phys.* **2008**, *128*, 044118

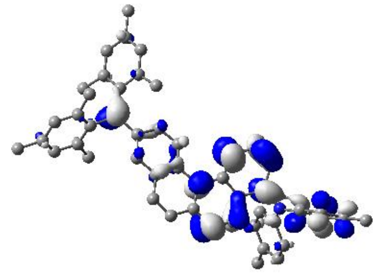

LUMO **1**$^{1-}$

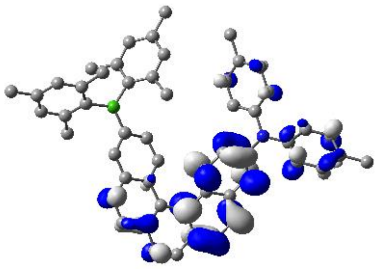

LUMO **2**$^{1-}$

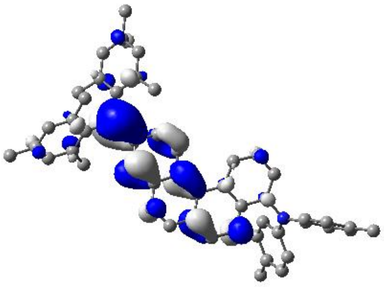

SOMO **1**$^{1-}$

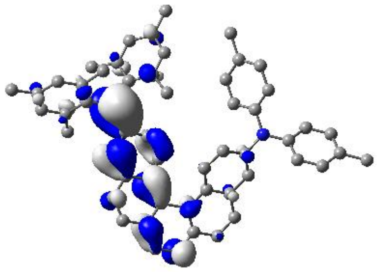

SOMO **2**$^{1-}$

SOMO/LUMO gap: 2.73 eV

SOMO/LUMO gap: 2.65 eV

Figure S13. Frontier molecular orbitals (SOMO and LUMO) of **1·K$_1$**, and **2·K$_1$** (as **1**$^{1-}$ and **2**$^{1-}$). H-atoms are omitted for clarity. Isovalue: ± 0.03 [e a$_0^{-3}$]^{½}.

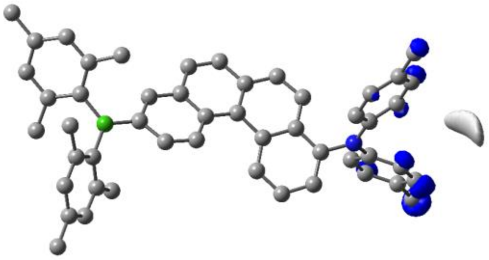

α-LUMO
E = +0.687 eV

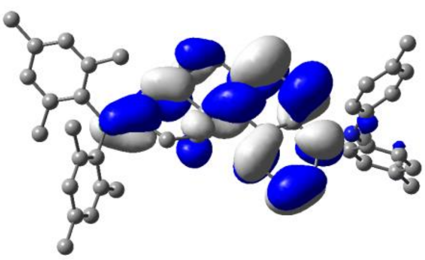

α-HOMO
E = -1.808 eV

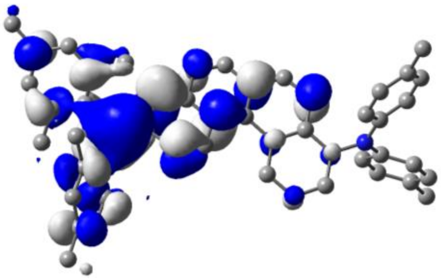

β-HOMO
E = -2.133 eV

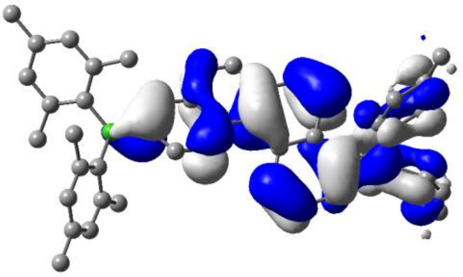

α-HOMO-1
E = -4.736 eV

Figure S14. Frontier molecular orbitals of **1**·**K**$_2$ (as **1**$^{2-}$: singlet (open-shell), SMD=Et_2O). H-atoms are omitted for clarity. Isovalue: ± 0.02 [e a_0^{-3}]^{½}.

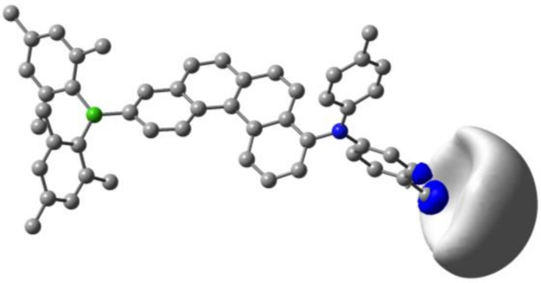

α-LUMO
E = +2.686 eV

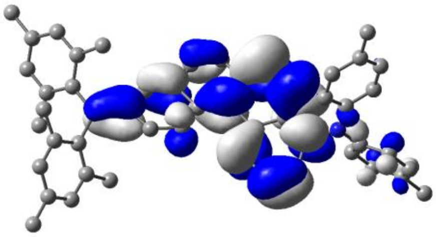

α-HOMO
E = +1.905 eV

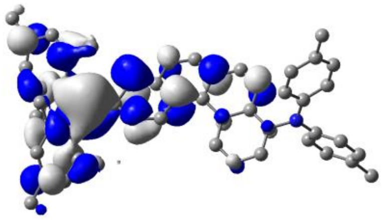

β-HOMO
E = +1.492 eV

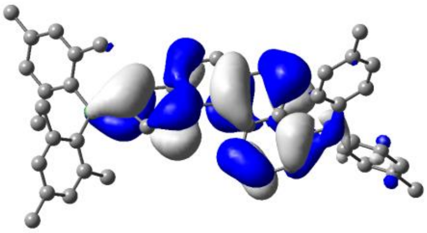

α-HOMO-1
E = -1.056 eV

Figure S15. Frontier molecular orbitals of **1·K$_2$** (as **1**$^{2-}$: singlet (open-shell), gas phase). H-atoms are omitted for clarity. Isovalue: ± 0.02 [*e* a_0^{-3}]^{½}. Δ$\mathbf{E}_{\alpha\beta}$ = 0.413 eV (39.85 kJ/mol).

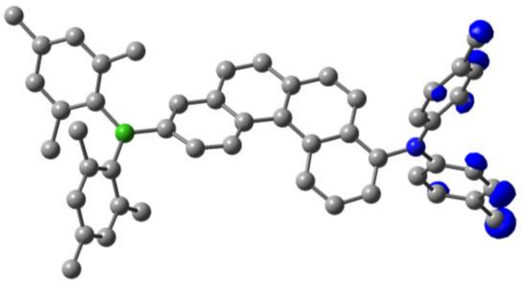

β-LUMO
E = +0.678 eV

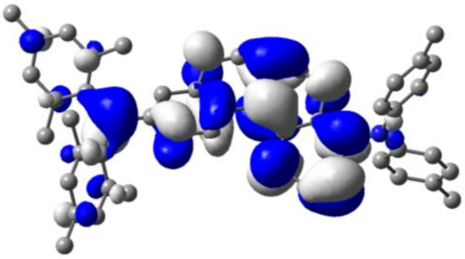

α-HOMO
E =-1.582 eV

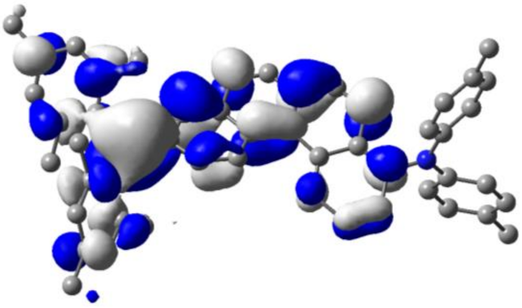

α-HOMO-1
E = -2.363 eV

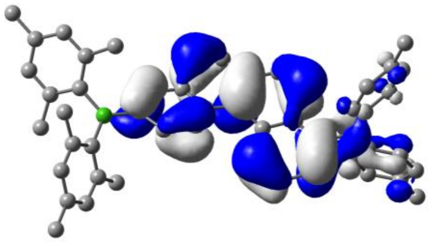

β-HOMO
E = -4.660 eV

Figure S16. Frontier molecular orbitals of **1**·$\mathbf{K_2}$ (as $\mathbf{1}^{2-}$: triplet, SMD=Et_2O). H-atoms are omitted for clarity. Isovalue: ± 0.02 [e a_0^{-3}]^{½}.

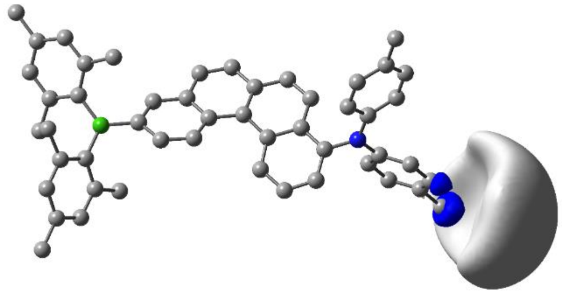

α-LUMO
E = +2.680 eV

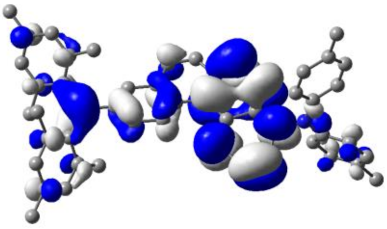

α-HOMO
E =+2.039 eV

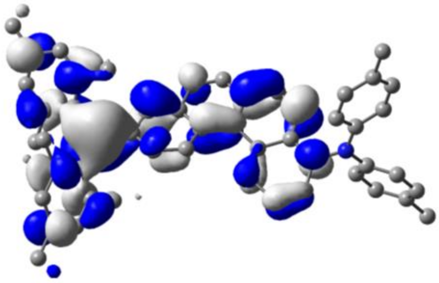

α-HOMO-1
E = +1.224 eV

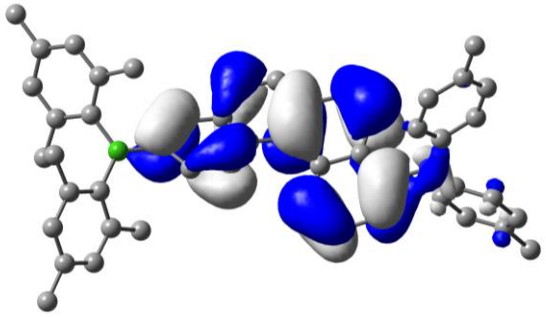

β-HOMO
E = -1.080 eV

Figure S17. Frontier molecular orbitals of **1**·$\mathbf{K_2}$ (as $\mathbf{1}^{2-}$: triplet, gas phase). H-atoms are omitted for clarity. Isovalue: ± 0.02 [e a_0^{-3}]^{½}.

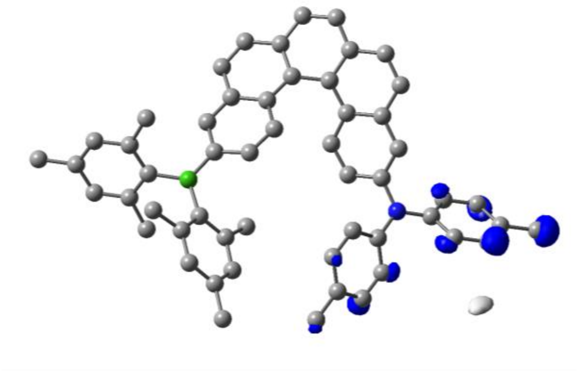

α-LUMO
E = +0.628 eV

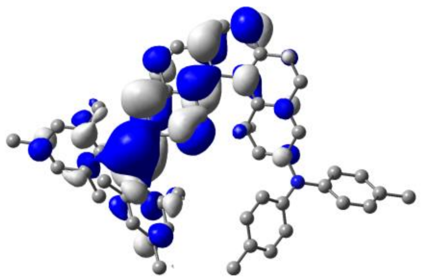

α-HOMO
E = -2.070 eV

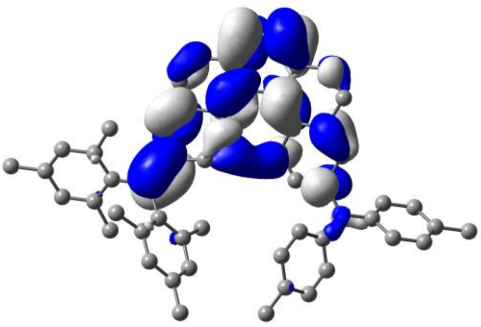

β-HOMO
E = -1.828 eV

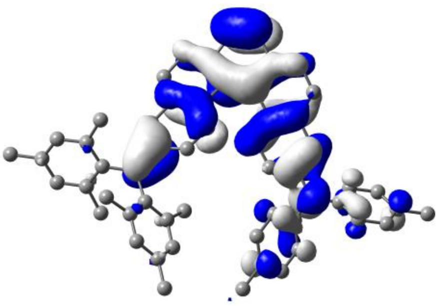

α-HOMO-1
E = -4.641 eV

Figure S18. Frontier molecular orbitals of **2**·**K**$_2$ (as **2**$^{2-}$: singlet (open-shell), SMD=Et$_2$O). H-atoms are omitted for clarity. Isovalue: ± 0.02 [e a_0^{-3}]^{½}.

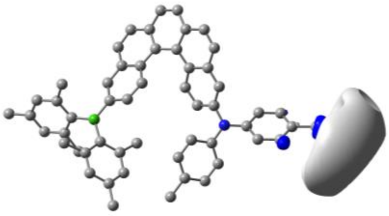

α-LUMO
E = +2.603 eV

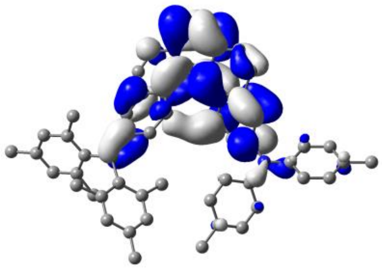

α-HOMO
E = +1.875 eV

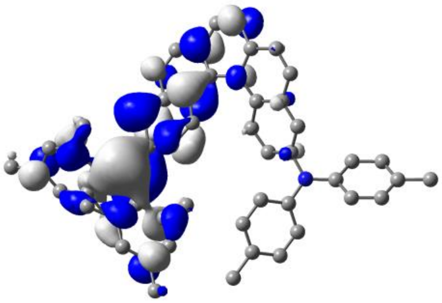

β-HOMO
E = +1.482 eV

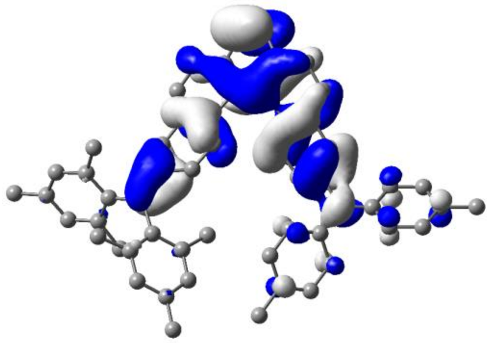

β-HOMO-1
E = -1.034 eV

Figure S19. Frontier molecular orbitals of **2·K₂** (as **2**$^{2-}$: singlet (open-shell), gas phase). H-atoms are omitted for clarity. Isovalue: ± 0.02 [*e* a_0^{-3}]^{½}. Δ$\mathbf{E_{\alpha\beta}}$ = 0.393 eV (37.92 kJ/mol).

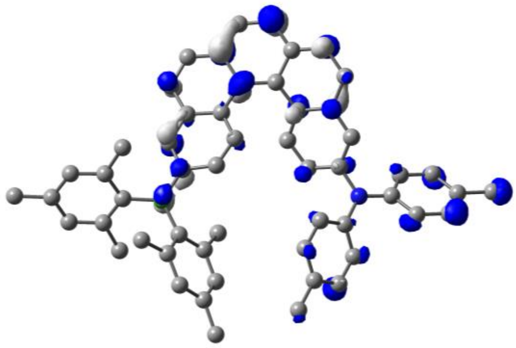

β-LUMO
E = +0.662 eV

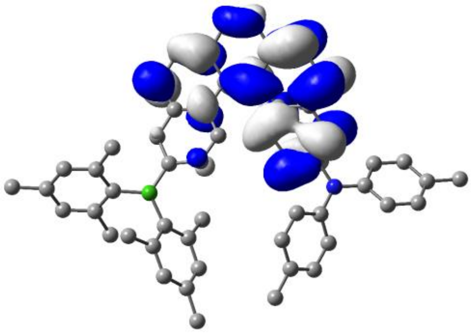

α-HOMO
E = -1.860 eV

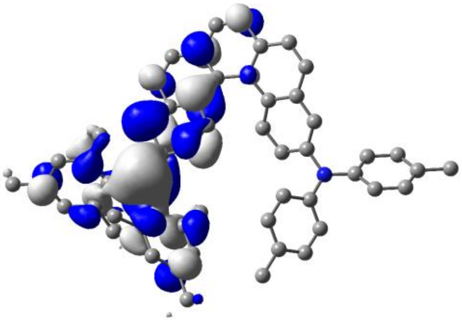

α-HOMO-1
E = -2.326 eV

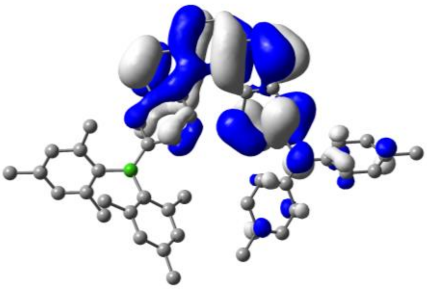

β-HOMO
E = -4.699 eV

Figure S20. Frontier molecular orbitals of **2·K$_2$** (as **2**$^{2-}$: triplet, SMD=Et_2O). H-atoms are omitted for clarity. Isovalue: ± 0.02 [e a_0^{-3}]^{½}.

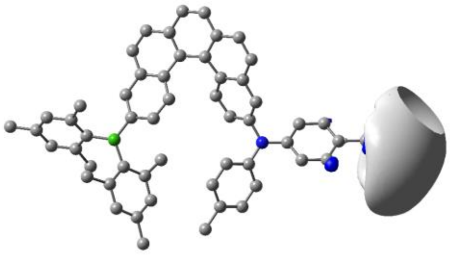

α-LUMO
E = +2.691 eV

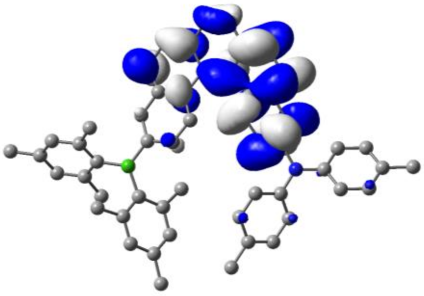

α-HOMO
E =+1.871 eV

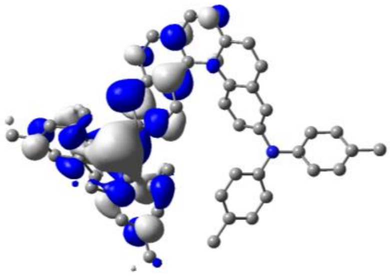

α-HOMO-1
E = +1.302 eV

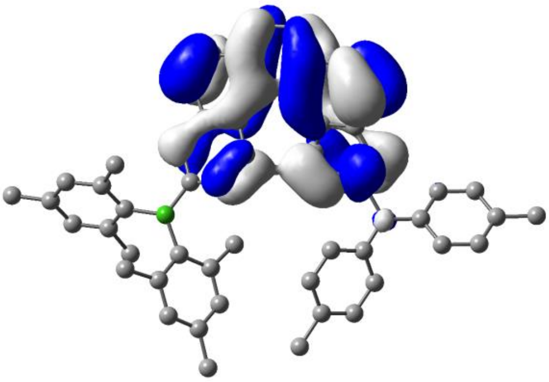

β-HOMO
E = -1.019 eV

Figure S21. Frontier molecular orbitals of **2·K$_2$** (as **2**$^{2-}$: triplet, gas phase). H-atoms are omitted for clarity. Isovalue: ± 0.02 [e a_0^{-3}]^{½}.

Table S4. Comparison between experimental and calculated geometric parameters of **1** and corresponding anion and dianion.

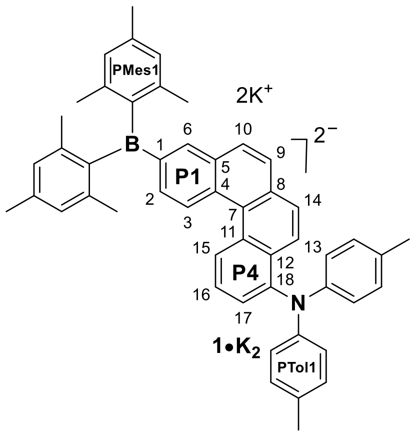

Compound	$\mathbf{1}^{a}$	$\mathbf{1{\cdot}K_1}^{a}$	$\mathbf{1}^{2-}\mathbf{\cdot(cs)}^{b}$	$\mathbf{1}^{2-}\mathbf{\cdot(os)}^{c}$	$\mathbf{1}^{2-}$ **(triplet)**^{d}
C3-C15	3.009(3)	3.037(3)	3.048	3.052	3.056
C15-C16	1.367(3)	1.377(3)	1.401	1.409	1.409
C13-C14	1.347(2)	1.359(3)	1.401	1.398	1.389
C9–C10	1.354(2)	1.356(3)	1.353	1.355	1.356
C2–C3	1.366(2)	1.368(3)	1.364	1.377	1.385
B–C_{Hel}	1.557(3)	1.533(3)	1.497	1.544	1.576
B–C_{Mes}	1.575(3)	1.598(3)	1.605	1.593	1.583
B–C_{Mes}	1.575(3)	1.601(3)	1.604	1.592	1.584
N–C_{Hel}	1.428(2)	1.447(2)	1.434	1.438	1.437
N–C_{Mes}	1.405(2)	1.434(3)	1.405	1.406	1.406
N–C_{Mes}	1.430(2)	1.398(3)	1.398	1.398	1.397
∠P1$_{Hel}$-BC_3	24.30(6)	4.95(9)	12.09	19.32	29.07
∠PMes1-BC_3	51.41(6)	60.86(8)	52.97	49.78	46.15
∠PMes2-BC_3	54.99(6)	63.78(8)	50.71	48.26	45.32
∠P4$_{Hel}$-NC_3	66.42(6)	77.18(8)	67.88	67.10	68.46
∠PTol1-NC_3	7.99(7)	14.90(9)	19.49	18.26	18.86
∠PTol2-NC_3	58.04(6)	57.82(9)	38.36	39.62	39.01
∠P1$_{Hel}$-P4$_{Hel}$	29.88(5)	32.04(7)	29.31	28.53	27.60
	121.36(16)	120.48(16)	121.67	120.83	119.84
∠C–B–C	119.65(16)	119.51(18)	121.24	120.50	119.65
	118.96(15)	119.97(18)	117.08	118.67	120.50
	122.69(14)	121.91(16)	119.82	120.16	120.15
∠C–N–C	121.76(14)	119.28(16)	122.77	122.59	122.54
	114.95(14)	114.32(16)	117.30	117.17	117.29
Sum ∠C–B–C	359.97(16)	359.96(18)	359.99	360.00	359.99
Sum ∠C–N–C	359.40(14)	355.51(16)	359.89	359.92	359.98

cs = closed-shell, os = open-shell. [a] Experimental values. [b] M062X/6-31G++(d)/gasphase; $\langle S^2 \rangle = 0.0000$. [c] UM062X/6-31G++(d)/gasphase guess=mix; $\langle S^2 \rangle = 0.8097$. [d] UM062X/6-31G++(d)/gasphase triplet; $\langle S^2 \rangle = 2.0203$.

Table S5. Comparison between experimental and calculated geometric parameters of **2** and corresponding anion and dianion.

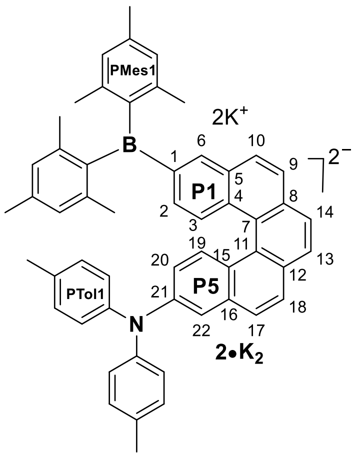

Compound	**2**[a]	**2·K$_1$ / 2·K$_2$**[a,b]	**2^{2-}·(cs)**[c]	**2^{2-}·(os)**[d]	**2^{2-} (triplet)**[e]
C3–C19	2.8629(18)	2.949(5)	2.876	2.896	2.939
C2–C3	1.367(3)	1.360(4)	1.362	1.370	1.373
C9-C10	1.351(3)	1.353(4)	1.356	1.362	1.370
C7-C11	1.443(2)	1.474(4)	1.471	1.470	1.433
C13-C14	1.356(3)	1.362(6)	1.405	1.397	1.362
C17-C18	1.349(2)	1.336(6)	1.361	1.359	1.400
C19-C20	1.367(2)	1.368(6)	1.372	1.374	1.401
B–C$_{Hel}$	1.555(3)	1.520(4)	1.496	1.537	1.553
B–C$_{Mes}$	1.585(2)	1.597(4)	1.605	1.595	1.591
B–C$_{Mes}$	1.579(2)	1.605(4)	1.606	1.597	1.595
N–C$_{Hel}$	1.411(2)	1.420(4)	1.433	1.433	1.438
N–C$_{Mes}$	1.432(2)	1.436(4)	1.399	1.398	1.398
N–C$_{Mes}$	1.412(2)	1.419(4)	1.411	1.410	1.409
∠P1$_{Hel}$-BC$_3$	12.80(5)	12.53(12)	12.27	16.87	19.42
∠PMes1-BC$_3$	64.58(5)	60.28(11)	53.86	51.79	51.40
∠PMes2-BC$_3$	62.89(5)	57.19(11)	50.34	47.76	46.67
∠P5$_{Hel}$-NC$_3$	29.24(5)	32.19(13)	49.84	49.92	51.64
PTol1-NC$_3$	29.60(6)	27.38(13)	28.71	29.96	29.68
PTol2-NC$_3$	53.35(7)	65.44(12)	32.63	30.72	31.11
∠P1$_{Hel}$-P5$_{Hel}$	45.09(4)	39.74(10)	42.53	44.50	43.41
∠C–B–C	121.33(11)	120.4(2)	122.51	121.89	122.32
	118.21(12)	119.3(2)	120.96	120.22	119.60
	120.44(12)	120.2(2)	116.51	117.87	118.05
∠C–N–C	119.61(11)	117.2(2)	122.20	122.24	122.59
	122.63(11)	122.4(2)	119.55	119.69	119.35
	117.74(11)	115.9(2)	118.10	118.02	118.01
Sum ∠ C–B–C	359.98(12)	359.9(2)	359.98	359.98	359.97
Sum ∠ C–N–C	359.98(11)	355.5(2)	359.85	359.95	359.95

cs = closed shell, os = open shell. [a] Experimental values. [b] The B(Mes)$_2$ group is disordered. Hence, values are given for both parts. [c] M062X/6-31G++(d)/gasphase; $\langle S^2 \rangle = 0.0000$. [d] UM062X/6-31G++(d)/gasphase guess=mix; $\langle S^2 \rangle = 0.8048$. [e] UM062X/6-31G++(d)/gasphase triplet; $\langle S^2 \rangle = 2.0340$.

Table S6. Expectations values, energies and singlet-triplet gaps of the lowest states for the dianions **1·K**$_2$ and **2·K**$_2$ (as **1**$^{2-}$ and **2**$^{2-}$) in gas phase.

			<S^2>	E (eV)	ΔE$_{ST}$ (eV)
**1**$^{2-}$	M062X/6-31G++(d)/ gasphase	-	0.0000	-54746.062393	
	UM062X/6-31G++(d)/gasphase	guess=mix	0.8097	-54746.169326	
	UM062X/6-31G++(d)/gasphase	triplet (sp)	2.0183	-54746.013837	0.16
	UM062X/6-31G++(d)/gasphase	triplet (opt)	2.0203	-54746.044407	0.13
**2**$^{2-}$	M062X/6-31G++(d)/ gasphase	-	0.0000	-58925.377397	
	UM062X/6-31G++(d)/gasphase	guess=mix	0.8048	-58925.463827	
	UM062X/6-31G++(d)/gasphase	triplet (sp)	2.0223	-58925.296766	0.17
	UM062X/6-31G++(d)/gasphase	triplet (opt)	2.0340	-58925.424384	0.04

Table S7. Expectations values, energies and singlet-triplet gaps of the lowest states for the dianions **1·K**$_2$ and **2·K**$_2$ (as **1**$^{2-}$ and **2**$^{2-}$) in Et_2O.

			<S^2>	E (eV)	ΔE$_{ST}$ (eV)
**1**$^{2-}$	M062X/6-31G++(d)/SMD=Et_2O	-		-54751.039923	0.02
	UM062X/6-31G++(d)/SMD=Et_2O	guess=mix	0.3188	-54751.054994	
	UM062X/6-31G++(d)/SMD=Et_2O	triplet (sp)	2.0193	-54750.567101	0.49
	UM062X/6-31G++(d)/SMD=Et_2O	triplet (opt)	2.0261	-54750.885852	0.17
**2**$^{2-}$	M062X/6-31G++(d)/SMD=Et_2O	-		-58930.416419	0.05
	UM062X/6-31G++(d)/SMD=Et_2O	guess=mix	0.3950	-58930.461623	
	UM062X/6-31G++(d)/SMD=Et_2O	triplet (sp)	2.0255	-58930.063597	0.40
	UM062X/6-31G++(d)/SMD=Et_2O	triplet (opt)	2.0355	-58930.338979	0.12

References

1 X. Jia, J. Nitsch, L. Ji, Z. Wu, A. Friedrich, F. Kerner, M. Moos, C. Lambert, T. B. Marder, *Chem. Eur. J.* 2019, **25**, 10845-10857.

2 S. V. Rosokha, J. K. Kochi, *J. Org. Chem.* 2006, **71**, 9357-9365.

3 G. M. Sheldrick, *Acta Crystallogr.* 2015, **A71**, 3-8.

4 G. M. Sheldrick, *Acta Crystallogr.* 2008, **A64**, 112-122.

5 C. B. Hübschle, G. M. Sheldrick, B. Dittrich, *J. Appl. Crystallogr.* 2011, **44**, 1281-1284.

6 C. F. Macrae, I. J. Bruno, J. A. Chisholm, P. R. Edgington, P. McCabe, E. Pidcock, L. Rodriguez-Monge, R. Taylor, J. van de Streek, P. A. Wood, *J. Appl. Crystallogr.* 2008, **41**, 466-470.

7 K. Brandenburg, Diamond (version 4.4.0), Crystal and Molecular Structure Visualization, Crystal Impact H. Putz & K. Brandenburg GbR, Bonn (Germany), 2017.

8 S. Stoll, A. Schweiger, *J. Magn. Reson.* 2006, **178**, 42-55.

9 M. J. Frisch, G. W. Trucks, H. B. Schlegel, G. E. Scuseria, M. A. Robb, J. R. Cheeseman, G. Scalmani, V. Barone, G. A. Petersson, H. Nakatsuji, X. Li, M. Caricato, A. V. Marenich, J. Bloino, B. G. Janesko, R. Gomperts, B. Mennucci, H. P. Hratchian, J. V. Ortiz, A. F. Izmaylov, J. L. Sonnenberg, Williams, F. Ding, F. Lipparini, F. Egidi, J. Goings, B. Peng, A. Petrone, T. Henderson, D. Ranasinghe, V. G. Zakrzewski, J. Gao, N. Rega, G. Zheng, W. Liang, M. Hada, M. Ehara, K. Toyota, R. Fukuda, J. Hasegawa, M. Ishida, T. Nakajima, Y. Honda, O. Kitao, H. Nakai, T. Vreven, K. Throssell, J. A. Montgomery Jr., J. E. Peralta, F. Ogliaro, M. J. Bearpark, J. J. Heyd, E. N. Brothers, K. N. Kudin, V. N. Staroverov, T. A. Keith, R. Kobayashi, J. Normand, K. Raghavachari, A. P. Rendell, J. C. Burant, S. S. Iyengar, J. Tomasi, M. Cossi, J. M. Millam, M. Klene, C. Adamo, R. Cammi, J. W. Ochterski, R. L. Martin, K. Morokuma, O. Farkas, J. B. Foresman, D. J. Fox, Wallingford, Gaussian, Inc., CT, 2016.

10 Y. Zhao, D. G. Truhlar, *Theor. Chem. Acc.* 2007, **120**, 215-241.

11 (a) G. A. Petersson, A. Bennett, T. G. Tensfeldt, M. A. Al-Laham, W. A. Shirley, J. Mantzaris, *J. Chem. Phys.* 1988, **89**, 2193; (b) G. A. Petersson, M. A. AlLaham, *J. Chem. Phys.* 1991, **94**, 6081-6090.

12 T. Clark, J. Chandrasekhar, G. W. Spitznagel, P. V. R. Schleyer, *J. Comput. Chem.* 1983, **4**, 294-301.

13 A. V. Marenich, C. J. Cramer, D. G. Truhlar, *J. Phys. Chem. B* 2009, **113**, 6378-6396.

14 T. Lu, F. Chen, *J. Comput. Chem.* 2012, **33**, 580-592.

XYZ-coordinates (Å)

1·K$_1$ (as 1^{1-}, doublet, gasphase) E = -54769.79 eV

C	10.75869857	5.44398662	4.87043424
N	10.84634652	6.12649012	3.64374277
B	2.63545678	12.76027543	3.73921543
C	9.50548223	5.10800167	5.40500743
H	8.60571845	5.36487118	4.85446783
C	8.74569329	10.53729558	5.18385862
C	9.41245985	4.47177886	6.63693391
H	8.42614024	4.23180856	7.02974610
C	10.54794474	4.13504897	7.37994163
C	11.78982939	4.48040265	6.84359901
H	12.69541375	4.25297359	7.40291946

1·K$_2$ (as 1^{2-}, closed-shell S$_0$, gasphase) E = -54746.06 eV

C	10.75749269	5.33510348	4.90500178
N	10.84300281	6.06599902	3.71133219
B	2.61265006	12.80243509	3.73374664
C	9.50409996	5.10409587	5.51021659
H	8.61458942	5.50874622	5.04160778
C	8.80650279	10.61264486	5.10258209
C	9.40553874	4.38158149	6.69427048
H	8.41929555	4.23221434	7.13194859
C	10.53236502	3.84987487	7.33803402
C	11.77514668	4.10204119	6.74783465
H	12.68008306	3.72940225	7.22792108
C	11.89768058	4.83332628	5.56710595

C	11.90283477	5.13088690	5.61807112
H	12.88288225	5.41020056	5.24290724
C	10.42649004	3.42874041	8.70735546
H	11.39539729	3.37161743	9.21284864
H	10.05483796	2.40477058	8.58275882
H	9.73055369	3.95047863	9.37313399
C	11.96535177	6.01316829	2.79410159
C	12.66010609	4.80738913	2.64280292
H	12.33908775	3.93191439	3.19980498
C	8.22868648	8.83087024	3.49534663
C	13.75180955	4.72650082	1.78064897
H	14.27458915	3.77729572	1.67961128
C	8.49279149	11.80863688	5.79269443
H	9.25072879	12.21949147	6.45739100
C	14.17581975	5.82532170	1.03189202
C	13.46434205	7.02201202	1.17938172
H	13.76862506	7.89821958	0.60969880
C	12.38365442	7.12409094	2.04546085
H	11.84599510	8.06263077	2.15012243
C	15.35358379	5.73708896	0.09322734
H	15.77645420	4.72792560	0.08564872
H	16.14945462	6.43130209	0.38640436
C	9.73750243	6.91523050	3.19733365
C	9.07015181	6.54354365	2.04783412
H	9.38785269	5.64901375	1.51759917
C	8.03601855	7.35350071	1.55454174
H	7.55762284	7.10910057	0.61052750
C	7.62691109	8.46535095	2.26758054
H	6.85889133	9.10302832	1.84795659
C	9.38054004	8.09279091	3.91308463
C	9.91623040	9.81171991	5.51963838
H	10.58565158	10.24096674	6.26327916
C	10.20176561	8.59121079	4.96896241
H	11.08051200	8.03229293	5.27381493
C	6.30211368	11.91560704	4.73737964
C	7.34767458	12.49237892	5.53856293
H	7.17268079	13.47087718	5.98180756
C	3.97254491	12.03343778	3.89670511
C	5.08315712	12.58141294	4.57800415
H	4.99124763	13.57658081	5.01494648
C	4.17743816	10.69347864	3.42363010
H	3.32752222	10.17062390	2.98470488
C	5.36687084	10.02341827	3.53820643
H	5.40760643	8.98487162	3.22792232
C	7.80102501	9.99669297	4.26667611
C	6.52040829	10.62317041	4.14483144

H	12.88375365	5.03044705	5.16185974
C	10.40599083	3.03457125	8.60431422
H	11.35878261	2.98873833	9.14602276
H	10.09902182	1.99641488	8.40430941
H	9.65799005	3.45948980	9.28583488
C	11.98745852	6.03334254	2.88147772
C	12.64890092	4.83044342	2.56105466
H	12.29004583	3.89554758	2.97968740
C	8.23712570	8.82743747	3.47099680
C	13.75103304	4.83454896	1.70181425
H	14.24021602	3.88891198	1.46772785
C	8.55438085	11.90931343	5.66684644
H	9.33539789	12.35484030	6.28503311
C	14.22560459	6.01142598	1.11918658
C	13.54700870	7.20729322	1.42970457
H	13.88699968	8.14515718	0.99274217
C	12.45585066	7.22223454	2.29090852
H	11.94793819	8.15221836	2.52572653
C	15.41401625	6.01146217	0.18681464
H	15.81076287	4.99870568	0.04775461
H	16.23545328	6.63702551	0.56624063
C	9.72660979	6.86176847	3.25272658
C	9.02179983	6.43545306	2.12747355
H	9.31258373	5.50252961	1.64790737
C	7.98898104	7.21760101	1.61823203
H	7.46349004	6.91743537	0.71431369
C	7.61424682	8.39302364	2.28860975
H	6.83108534	9.00329175	1.85711935
C	9.41430631	8.09570720	3.91559178
C	9.96551887	9.90665632	5.43396908
H	10.66157952	10.36401268	6.13860092
C	10.24339387	8.63845505	4.92187472
H	11.13545627	8.10223540	5.22922591
C	6.30743307	11.96571293	4.70645667
C	7.38710913	12.56894240	5.45113438
H	7.22096959	13.55777751	5.87911987
C	3.94324952	12.08491498	3.88849890
C	5.09173974	12.62308332	4.55820601
H	5.02104914	13.62999189	4.97136399
C	4.16775585	10.73716672	3.42160856
H	3.31867055	10.19810222	3.00177721
C	5.36112995	10.06843078	3.51825983
H	5.39367065	9.03099931	3.20243067
C	7.81269480	10.03169180	4.21599745
C	6.53825633	10.64980480	4.11901384
C	1.87447736	15.34072811	3.96824328
C	2.29250066	14.12187908	4.58917277
C	2.09243677	15.32249796	6.73524071
H	2.16885990	15.29965152	7.82396892

C	1.89238610	15.26473228	4.00115283
C	2.31142818	14.05572086	4.61482110
C	2.03466229	15.18078545	6.77065288
H	2.07652359	15.13218153	7.85953365
C	1.63878867	16.36746538	6.16149572
C	1.57320297	16.38659964	4.76673732
H	1.26336741	17.30302843	4.26240726
C	2.76433687	12.80333993	6.80687892
H	3.85018772	12.74644686	6.95091483
H	2.48083912	11.88598899	6.28363862
H	2.29361916	12.80784174	7.79648456
C	2.37201404	14.04097904	6.02909759
C	-0.24724238	12.25867736	4.51381398
H	-0.27144345	13.32480299	4.76734826
H	0.44050935	11.78402971	5.22155429
H	-1.24838022	11.84472989	4.67663884
C	-1.49842264	10.93715942	-0.16024052
H	-2.42997505	11.50323308	-0.04756688
H	-1.74009415	9.87787659	-0.00809653
H	-1.15460760	11.05315783	-1.19319644
C	3.24616463	12.25642000	0.77233854
H	3.76506233	13.06774831	1.29002351
H	3.19506827	12.49308050	-0.29643348
H	3.87857604	11.36820736	0.88944013
C	1.85724489	12.02793370	1.32921910
C	0.86813291	11.62045224	0.42464777
H	1.13854889	11.47686645	-0.62220929
C	-0.44880671	11.40489491	0.81802317
C	-0.76483601	11.62290424	2.16050999
H	-1.79047156	11.46447587	2.49672967
C	0.19522163	12.03840453	3.08384632
C	1.54472598	12.24479943	2.69376580
C	1.77961402	15.37478914	2.49652848
H	1.65242383	16.41960368	2.19270040
H	0.92789640	14.79980705	2.11500049
H	2.67237394	14.97870203	2.00098169
C	1.30598700	17.59731347	6.97036241
H	2.09908450	18.35128744	6.89257537
H	1.18546074	17.35139973	8.03037974
H	0.37727290	18.06423932	6.62330776
H	15.06412574	5.98713881	-0.93363623

C	1.69199708	16.50550905	6.11292953
C	1.59110196	16.48625118	4.71716673
H	1.29636015	17.39870553	4.19462290
C	2.77297618	12.93202943	6.81825406
H	3.85267305	12.74222345	6.78494040
H	2.30759599	12.02801779	6.41072043
H	2.47732104	13.04426226	7.87025796
C	2.38685440	14.15605461	6.01554255
C	-0.29963663	12.26473396	4.52913602
H	-0.25084547	13.31934966	4.82035408
H	0.34706035	11.72428396	5.23076598
H	-1.33149408	11.91746874	4.67525634
C	-1.54617306	10.76013365	-0.09413667
H	-2.52854569	11.22437074	0.07030409
H	-1.69072993	9.67295910	0.01128855
H	-1.26313335	10.94297095	-1.13874699
C	3.17652715	12.22525082	0.74240941
H	3.63839638	13.12312894	1.16564221
H	3.11854554	12.33053196	-0.34973814
H	3.87188263	11.40689525	0.96507902
C	1.80170229	11.98518867	1.33210978
C	0.81232325	11.52016416	0.45486408
H	1.07996558	11.33902821	-0.58793092
C	-0.50434076	11.29741758	0.85984246
C	-0.81148809	11.57306728	2.19674400
H	-1.83079470	11.40653885	2.55098907
C	0.14836091	12.04016605	3.09913104
C	1.50707551	12.25872086	2.70623194
C	1.76829253	15.45122165	2.46081542
H	1.56890528	16.48761841	2.15607654
H	0.97302152	14.81742092	2.05383905
H	2.69676236	15.12298173	1.97809715
C	1.35465760	17.74554100	6.90876644
H	1.79255581	18.64964070	6.46232458
H	1.72774910	17.67179581	7.93794327
H	0.26890232	17.92250972	6.97172986
H	15.15654734	6.40338571	-0.80782592

1·K$_2$ (as 1^{2-}, open-shell S$_0$, gasphase) E = -54746.19 eV

C	10.7412713	5.3727102	4.92213804
N	10.8033931	6.00308297	3.6763458

1·K$_2$ (as 1^{2-}, triplet, gasphase) E = -54746.04 eV

C	10.7989043	5.39882224	5.01206731
N	10.7862813	5.98050589	3.74159578

B	2.63540268	12.7684059	3.70355465
C	9.4926202	5.17363625	5.54259637
H	8.59751535	5.52226114	5.03748492
C	8.82220962	10.5521007	5.01626078
C	9.40948556	4.56589084	6.787466
H	8.42735481	4.43633357	7.24007495
C	10.546284	4.13198256	7.47919417
C	11.7820358	4.35798536	6.87298978
H	12.6937489	4.06203657	7.39099265
C	11.8892468	4.97255266	5.62687906
H	12.8720753	5.16217654	5.20778533
C	10.4311962	3.46557813	8.82822536
H	11.4204916	3.29074269	9.26486883
H	9.92262509	2.4956042	8.75924229
H	9.86011026	4.08252627	9.53190685
C	11.9494293	5.96224499	2.86350274
C	12.6853549	4.78413854	2.66745517
H	12.3801831	3.8753167	3.17844455
C	8.24004035	8.77020203	3.39953537
C	13.7910941	4.77233817	1.8161391
H	14.3424316	3.84274907	1.6788071
C	8.5836425	11.863088	5.56088447
H	9.36629697	12.3047539	6.17838627
C	14.1903738	5.91317914	1.12185104
C	13.4353793	7.08188821	1.30713666
H	13.7182429	7.98990197	0.77650586
C	12.3391524	7.11405356	2.15735193
H	11.7589365	8.02328755	2.29005223
C	15.3776552	5.9007602	0.19089466
H	15.8595796	4.91729742	0.18110745
H	16.1313793	6.63925711	0.49097685
C	9.69472998	6.78974162	3.20896457
C	8.99690225	6.3605231	2.07967771
H	9.28624039	5.42104901	1.61075647
C	7.98084732	7.14864297	1.55770207
H	7.45713579	6.84757154	0.65402961
C	7.61499233	8.33833534	2.21858355
H	6.8475472	8.95959483	1.77356127
C	9.39690913	8.02931948	3.85952607
C	9.97712087	9.84580241	5.36539052
H	10.6760434	10.304071	6.0644271
C	10.2291918	8.56468578	4.86469631
H	11.1092023	8.01172815	5.18278881
C	6.35550152	11.9280501	4.57767662
C	7.43265467	12.5363288	5.32032345
H	7.27299248	13.5342509	5.7279145

B	2.59524682	12.8008744	3.68779781
C	9.58588605	5.18993335	5.69697473
H	8.65927452	5.48805772	5.21684869
C	8.79118036	10.542797	4.94724213
C	9.57711812	4.63581798	6.96922955
H	8.62035808	4.49540601	7.47049169
C	10.7564617	4.26678562	7.62674974
C	11.9563981	4.50096381	6.9549461
H	12.8988701	4.25406067	7.44272264
C	11.9890709	5.0624442	5.68025733
H	12.9458315	5.25979141	5.20758517
C	10.7222002	3.66136459	9.00861972
H	11.7358265	3.5028078	9.39238774
H	10.2096374	2.69116765	9.01336506
H	10.195016	4.31066038	9.7176333
C	11.8859592	5.91384871	2.86838331
C	12.6380631	4.7413357	2.69713083
H	12.3865936	3.85838235	3.27789287
C	8.15117641	8.6804596	3.45417997
C	13.6906377	4.70111082	1.78230133
H	14.2546842	3.77616908	1.66636712
C	8.58634035	11.8846873	5.418866
H	9.3876761	12.3449261	5.99711337
C	14.0212766	5.80611182	0.99929843
C	13.2504479	6.96787258	1.16137241
H	13.479004	7.84806273	0.56210763
C	12.2053577	7.02795198	2.07220905
H	11.609064	7.92969086	2.18165534
C	15.1488848	5.76097549	-0.00201886
H	15.6682248	4.79733593	0.03349548
H	15.8893165	6.54713036	0.19090103
C	9.63322096	6.71147615	3.29417099
C	8.86754397	6.18728348	2.23847324
H	9.14398723	5.22466732	1.81196609
C	7.80214687	6.92213203	1.74962964
H	7.22225115	6.55062177	0.90743116
C	7.44822942	8.1491731	2.34489827
H	6.64890327	8.72562493	1.89849289
C	9.33974159	7.97735465	3.88265572
C	9.97174602	9.86402262	5.28536379
H	10.6959836	10.3622592	5.92852856
C	10.2150451	8.57655094	4.82404232
H	11.1193655	8.04952707	5.11768874
C	6.35056853	11.929629	4.46634979
C	7.44869912	12.5697714	5.14706009
H	7.3128753	13.5940595	5.49124411

C	3.98969215	12.0372879	3.82156279
C	5.13155543	12.591727	4.44148735
H	5.06328791	13.6017208	4.85265059
C	4.18893595	10.6962817	3.36458864
H	3.3309259	10.1641433	2.95013167
C	5.38796379	10.0251432	3.44854359
H	5.41933744	8.98774997	3.13416714
C	7.84093519	9.97650523	4.13500027
C	6.56152695	10.6163643	4.02051999
C	1.78510333	15.2286803	4.10001522
C	2.30664619	14.017362	4.63657393
C	2.11755789	15.0732589	6.84876505
H	2.22862257	14.992448	7.93204575
C	1.62864728	16.2619138	6.30709265
C	1.46403821	16.3116562	4.92213504
H	1.07872871	17.2261747	4.46639049
C	2.91287035	12.7196662	6.7615151
H	4.00041023	12.5928092	6.70924794
H	2.48937355	11.8250034	6.29156411
H	2.6197034	12.7453772	7.81810526
C	2.45289716	13.9730527	6.05322474
C	-0.28927579	12.3694172	4.42666718
H	-0.30590377	13.4356006	4.68475673
H	0.38994875	11.8914347	5.14140649
H	-1.29858803	11.9654024	4.57378859
C	-1.5056288	11.0693117	-0.26347385
H	-2.48955745	11.494003	-0.03085697
H	-1.61545003	9.97612554	-0.26792479
H	-1.23940487	11.3731225	-1.28271128
C	3.25287397	12.2893988	0.73338938
H	3.71990395	13.1663055	1.19492
H	3.22598772	12.425968	-0.35465101
H	3.91892131	11.4479657	0.95533916
C	1.85770019	12.0699853	1.27330461
C	0.86965292	11.6933549	0.35776406
H	1.1499007	11.5443325	-0.68696598
C	-0.46311241	11.5214248	0.72981431
C	-0.78775028	11.7598334	2.0663148
H	-1.82356277	11.6380921	2.39006483
C	0.17404816	12.1406262	3.00486784
C	1.54349553	12.2975395	2.64461775
C	1.56678197	15.3896621	2.61178934
H	1.40546516	16.4442859	2.35611369
H	0.69949657	14.8156371	2.26244122
H	2.42867368	15.0171236	2.04693191
C	1.31834065	17.4540259	7.17925264

C	3.96022254	12.0231088	3.81830016
C	5.12272915	12.6062443	4.33384383
H	5.07992136	13.6406194	4.68333349
C	4.10678997	10.6571928	3.46053111
H	3.22543363	10.1177012	3.1103493
C	5.30789096	9.97227885	3.54193233
H	5.31404773	8.91617393	3.29891807
C	7.79586008	9.91860694	4.12591556
C	6.50300984	10.5857495	4.00462029
C	1.73111435	15.2228639	4.23915041
C	2.26661383	13.9814831	4.69214019
C	2.13217896	14.9104653	6.96747527
H	2.2660022	14.7667743	8.04187638
C	1.63232251	16.1312337	6.50593947
C	1.43254576	16.2566251	5.13157242
H	1.0377554	17.1955123	4.7376849
C	2.90986424	12.5630579	6.72846221
H	3.98937489	12.4125886	6.61669474
H	2.43605809	11.7028267	6.24074499
H	2.66880801	12.5439793	7.7981873
C	2.4457038	13.8594286	6.10449968
C	-0.38798029	12.5003948	4.20334121
H	-0.39628867	13.5622745	4.48164016
H	0.22672184	11.994368	4.95709426
H	-1.41573952	12.1221353	4.27054073
C	-1.30688274	11.3033961	-0.58282692
H	-2.33807725	11.5127177	-0.27526928
H	-1.24352199	10.2306985	-0.81215066
H	-1.11953106	11.8439748	-1.51938252
C	3.40037708	12.3739005	0.76332616
H	3.83003323	13.271854	1.22320962
H	3.46120968	12.4687048	-0.32753358
H	4.0432996	11.5424357	1.07183535
C	1.96581663	12.1798585	1.19883023
C	1.03532125	11.8454588	0.21445784
H	1.38255352	11.7042214	-0.81132043
C	-0.32762589	11.7062139	0.49206508
C	-0.73483244	11.9391424	1.80493594
H	-1.79338859	11.843317	2.05552202
C	0.16934718	12.2805638	2.81516038
C	1.56580822	12.4009919	2.55298582
C	1.4792726	15.4753616	2.76989075
H	1.31131303	16.5437802	2.58508848
H	0.60528168	14.9231891	2.40069844
H	2.32832931	15.1388351	2.16338229
C	1.34184113	17.2669753	7.45591932

H	2.19191123	18.1100214	7.29653023
H	1.01045529	17.1409372	8.18393153
H	0.51066057	18.0606912	6.75263804
H	15.0833041	6.13861648	-0.83876873

1·K$_2$ (as 1^{2-}, closed-shell S$_0$, SMD=Et$_2$O) E = -54751.03 eV

C	10.6865109	5.31356105	4.82223622
N	10.8018918	6.01626615	3.61885975
B	2.68041758	12.7443885	3.73862975
C	9.41207255	5.06158311	5.36456712
H	8.53678422	5.42847083	4.83812048
C	8.84049201	10.543308	5.0892948
C	9.27626037	4.38056598	6.5661445
H	8.27501669	4.212353	6.960056
C	10.3839333	3.92070845	7.28737483
C	11.6447397	4.19770023	6.75897669
H	12.5339783	3.88477737	7.30509454
C	11.804096	4.88610943	5.55813968
H	12.8033218	5.11395365	5.20143238
C	10.2125158	3.16922538	8.58463068
H	11.175711	3.02827607	9.08678375
H	9.7743574	2.1761193	8.42278171
H	9.55188213	3.70782058	9.27369418
C	11.9795397	6.02421662	2.85290699
C	12.737471	4.86462549	2.63757623
H	12.4203886	3.93002017	3.09146006
C	8.26971317	8.81096429	3.41496635
C	13.8807814	4.90273313	1.83901325
H	14.4492478	3.98620383	1.68586796
C	8.59199842	11.8367506	5.67414988
H	9.37275097	12.2693981	6.30147697
C	14.2971682	6.07868123	1.21634677
C	13.52164	7.22915999	1.42076474
H	13.8178358	8.16378543	0.94674792
C	12.3880106	7.21230744	2.22129387
H	11.796763	8.11113828	2.37465139
C	15.5269248	6.12107537	0.34287847
H	16.01734	5.1425601	0.30628474
H	16.2588847	6.84810326	0.71532544
C	9.71226766	6.84014295	3.16356074
C	9.04882274	6.47603815	2.00139621
H	9.3470119	5.55958492	1.49408206
C	8.05346632	7.30407862	1.48193789
H	7.56388439	7.06011987	0.54319718
C	7.68430444	8.45308431	2.19332293

H	0.68054371	18.009042	6.9934555
H	2.25914034	17.7896009	7.76081415
H	0.85591628	16.9086638	8.37225095
H	14.7831275	5.90513808	-1.02627812

1·K$_2$ (as 1^{2-}, open-shell S$_0$, SMD=Et$_2$O) E = -54751.05 eV

C	10.7412713	5.3727102	4.92213804
N	10.8033931	6.00308297	3.6763458
B	2.63540268	12.7684059	3.70355465
C	9.4926202	5.17363625	5.54259637
H	8.59751535	5.52226114	5.03748492
C	8.82220962	10.5521007	5.01626078
C	9.40948556	4.56589084	6.787466
H	8.42735481	4.43633357	7.24007495
C	10.546284	4.13198256	7.47919417
C	11.7820358	4.35798536	6.87298978
H	12.6937489	4.06203657	7.39099265
C	11.8892468	4.97255266	5.62687906
H	12.8720753	5.16217654	5.20778533
C	10.4311962	3.46557813	8.82822536
H	11.4204916	3.29074269	9.26486883
H	9.92262509	2.4956042	8.75924229
H	9.86011026	4.08252627	9.53190685
C	11.9494293	5.96224499	2.86350274
C	12.6853549	4.78413854	2.66745517
H	12.3801831	3.8753167	3.17844455
C	8.24004035	8.77020203	3.39953537
C	13.7910941	4.77233817	1.8161391
H	14.3424316	3.84274907	1.6788071
C	8.5836425	11.863088	5.56088447
H	9.36629697	12.3047539	6.17838627
C	14.1903738	5.91317914	1.12185104
C	13.4353793	7.08188821	1.30713666
H	13.7182429	7.98990197	0.77650586
C	12.3391524	7.11405356	2.15735193
H	11.7589365	8.02328755	2.29005223
C	15.3776552	5.9007602	0.19089466
H	15.8595796	4.91729742	0.18110745
H	16.1313793	6.63925711	0.49097685
C	9.69472998	6.78974162	3.20896457
C	8.99690225	6.3605231	2.07967771
H	9.28624039	5.42104901	1.61075647
C	7.98084732	7.14864297	1.55770207
H	7.45713579	6.84757154	0.65402961
C	7.61499233	8.33833534	2.21858355
H	6.8475472	8.95959483	1.77356127

H	6.93187334	9.10820177	1.76871288
C	9.4084334	8.04277228	3.87158331
C	9.96360193	9.80283306	5.43590127
H	10.6547062	10.2325524	6.16258821
C	10.2212323	8.52642769	4.91852386
H	11.086261	7.9565174	5.24515733
C	6.34358578	11.9097066	4.70777266
C	7.42933684	12.4987195	5.46899808
H	7.26255774	13.4805803	5.91238707
C	3.99178889	12.0343966	3.87480214
C	5.13972359	12.5644261	4.56708076
H	5.061134	13.5644668	4.99946202
C	4.21956697	10.6920012	3.3803289
H	3.36791844	10.1614351	2.95047267
C	5.40627232	10.0251096	3.47159563
H	5.44577185	8.99143432	3.14069074
C	7.84064964	9.99376609	4.18910063
C	6.58186932	10.6031533	4.09634875
C	1.88958127	15.2535481	3.93868644
C	2.36342165	14.0748391	4.57765099
C	2.12579524	15.2887575	6.69932608
H	2.2038233	15.2847981	7.78849774
C	1.67703091	16.4413139	6.05929567
C	1.56369004	16.3987981	4.66799971
H	1.21511649	17.2865414	4.1358834
C	2.90058513	12.9287757	6.80680547
H	3.99234419	12.8703236	6.89390762
H	2.59636596	11.9946051	6.32478315
H	2.47879632	12.9765636	7.81863
C	2.46751103	14.1275094	5.9938194
C	-0.19639582	12.3460214	4.61961038
H	-0.20974897	13.4208939	4.83649556
H	0.52693227	11.8995197	5.31026808
H	-1.1911088	11.9385806	4.838963
C	-1.65987922	10.8961284	0.04703488
H	-2.57658486	11.4848384	0.17629596
H	-1.9197703	9.8457858	0.23582209
H	-1.3540731	10.9751237	-1.00235156
C	3.15022673	12.1134983	0.7539653
H	3.6990949	12.9295764	1.23343542
H	3.06080889	12.3104185	-0.32183629
H	3.77618105	11.2224531	0.88450795
C	1.7828105	11.9368076	1.37489961
C	0.74739019	11.5263311	0.52376206
H	0.97548255	11.3331788	-0.52637854
C	-0.5623206	11.3712062	0.96819196
C	9.39690913	8.02931948	3.85952607
C	9.97712087	9.84580241	5.36539052
H	10.6760434	10.304071	6.0644271
C	10.2291918	8.56468578	4.86469631
H	11.1092023	8.01172815	5.18278881
C	6.35550152	11.9280501	4.57767662
C	7.43265467	12.5363288	5.32032345
H	7.27299248	13.5342509	5.7279145
C	3.98969215	12.0372879	3.82156279
C	5.13155543	12.591727	4.44148735
H	5.06328791	13.6017208	4.85265059
C	4.18893595	10.6962817	3.36458864
H	3.3309259	10.1641433	2.95013167
C	5.38796379	10.0251432	3.44854359
H	5.41933744	8.98774997	3.13416714
C	7.84093519	9.97650523	4.13500027
C	6.56152695	10.6163643	4.02051999
C	1.78510333	15.2286803	4.10001522
C	2.30664619	14.017362	4.63657393
C	2.11755789	15.0732589	6.84876505
H	2.22862257	14.992448	7.93204575
C	1.62864728	16.2619138	6.30709265
C	1.46403821	16.3116562	4.92213504
H	1.07872871	17.2261747	4.46639049
C	2.91287035	12.7196662	6.7615151
H	4.00041023	12.5928092	6.70924794
H	2.48937355	11.8250034	6.29156411
H	2.6197034	12.7453772	7.81810526
C	2.45289716	13.9730527	6.05322474
C	-0.28927579	12.3694172	4.42666718
H	-0.30590377	13.4356006	4.68475673
H	0.38994875	11.8914347	5.14140649
H	-1.29858803	11.9654024	4.57378859
C	-1.5056288	11.0693117	-0.26347385
H	-2.48955745	11.494003	-0.03085697
H	-1.61545003	9.97612554	-0.26792479
H	-1.23940487	11.3731225	-1.28271128
C	3.25287397	12.2893988	0.73338938
H	3.71990395	13.1663055	1.19492
H	3.22598772	12.425968	-0.35465101
H	3.91892131	11.4479657	0.95533916
C	1.85770019	12.0699853	1.27330461
C	0.86965292	11.6933549	0.35776406
H	1.1499007	11.5443325	-0.68696598
C	-0.46311241	11.5214248	0.72981431
C	-0.78775028	11.7598334	2.0663148

C	-0.81958688	11.6537806	2.31204261
H	-1.83671711	11.5397786	2.69321382
C	0.18875933	12.069529	3.18269165
C	1.53628454	12.2168637	2.74669199
C	1.73139972	15.3054178	2.43498471
H	1.61019257	16.3405302	2.09218704
H	0.86166295	14.727591	2.09949365
H	2.60400048	14.8688801	1.93704234
C	1.3525942	17.697153	6.83186037
H	2.18809307	18.4101026	6.81440192
H	1.13880506	17.4697727	7.88253582
H	0.47873262	18.2107089	6.41287651
H	15.2784453	6.40895589	-0.68587822

H	-1.82356277	11.6380921	2.39006483
C	0.17404816	12.1406262	3.00486784
C	1.54349553	12.2975395	2.64461775
C	1.56678197	15.3896621	2.61178934
H	1.40546516	16.4442859	2.35611369
H	0.69949657	14.8156371	2.26244122
H	2.42867368	15.0171236	2.04693191
C	1.31834065	17.4540259	7.17925264
H	2.19191123	18.1100214	7.29653023
H	1.01045529	17.1409372	8.18393153
H	0.51066057	18.0606912	6.75263804
H	15.0833041	6.13861648	-0.83876873

1·K_2 (as 1^{2-}, triplet, SMD=Et_2O) E = -54750.89 eV

C	10.7744278	5.32757985	4.89260295
N	10.8452001	6.04578948	3.68496685
B	2.61725904	12.8112593	3.72304406
C	9.53625812	4.85819597	5.36280395
H	8.64231576	5.03784173	4.77290077
C	8.74556412	10.4776051	5.14338694
C	9.44918332	4.17834184	6.57279546
H	8.47464426	3.82740485	6.90847444
C	10.5776182	3.93302387	7.36411849
C	11.8029055	4.41635345	6.89771029
H	12.7000846	4.26524742	7.49559353
C	11.9063792	5.10997949	5.69351126
H	12.8718567	5.49464651	5.37727473
C	10.4600622	3.20172886	8.67794892
H	11.4467984	2.98131927	9.09718695
H	9.92398158	2.25305262	8.56032658
H	9.90907354	3.79690264	9.41597433
C	11.9774692	6.01808398	2.85197191
C	12.8193294	4.89865227	2.76210295
H	12.6099596	4.01643044	3.36010889
C	8.16356635	8.70835962	3.51779073
C	13.9145974	4.90089681	1.89959846
H	14.5455195	4.01499117	1.85053351
C	8.52788896	11.7860938	5.67030128
H	9.30060263	12.2114169	6.3107888
C	14.2091384	5.99846886	1.08703437
C	13.3534233	7.10435422	1.16919154
H	13.5459961	7.97714462	0.5472107
C	12.2628486	7.12314864	2.03017962
H	11.6128538	7.99259421	2.07174622
C	15.3958268	6.00307397	0.15597432
H	15.8981259	5.03058765	0.15228314

H	16.1331186	6.75883328	0.45258364
C	9.69411127	6.78776848	3.25062824
C	8.95450298	6.3026454	2.14991985
H	9.25536094	5.37545981	1.66739478
C	7.87615865	7.05118693	1.69856583
H	7.31986779	6.72492201	0.82136622
C	7.48170194	8.22195672	2.3550939
H	6.67564476	8.80336737	1.92724056
C	9.35770096	7.98931861	3.90771495
C	9.92674087	9.77896018	5.46522203
H	10.6291472	10.2408425	6.15899901
C	10.2076219	8.54502833	4.92227005
H	11.1203766	8.02034294	5.19094143
C	6.33407138	11.899038	4.6180352
C	7.40645601	12.5029797	5.37388164
H	7.26367113	13.5101752	5.76153372
C	3.95971934	12.0272335	3.88452405
C	5.11657198	12.5860255	4.46087475
H	5.07575368	13.611831	4.83353716
C	4.11088765	10.6655961	3.49673292
H	3.24149758	10.1311762	3.11137183
C	5.30927265	9.97510201	3.60201305
H	5.31733461	8.92513841	3.32990738
C	7.78344114	9.8861109	4.2451902
C	6.49047337	10.5688729	4.11244992
C	1.82985766	15.3028578	4.03195364
C	2.31707849	14.0954325	4.61218008
C	2.16533895	15.2361117	6.78828046
H	2.27273699	15.1903983	7.87343149
C	1.71958707	16.4231603	6.20362511
C	1.54996919	16.4260763	4.81749712
H	1.19319258	17.3355869	4.33185389
C	2.87557679	12.8559531	6.78851748
H	3.96521391	12.7312111	6.80810497
H	2.47313077	11.9496614	6.32138652
H	2.52760273	12.9022413	7.82684976
C	2.45942955	14.0968982	6.03146788
C	-0.30966261	12.4306128	4.43921448
H	-0.32814708	13.5011533	4.68042107
H	0.35773552	11.9593532	5.17111995
H	-1.32128509	12.0358119	4.58784211
C	-1.50362348	11.0756131	-0.24605621
H	-2.50506489	11.4175218	0.03711911
H	-1.53950789	9.98065805	-0.31784609
H	-1.28620446	11.4591429	-1.24928552
C	3.26209057	12.2490976	0.77452841

H	3.74075633	13.1342761	1.21077128
H	3.25498558	12.3549135	-0.31606922
H	3.91090804	11.4012762	1.02426242
C	1.85784156	12.0632775	1.30294985
C	0.87361367	11.6797684	0.38568241
H	1.16016115	11.5069322	-0.65306716
C	-0.46587383	11.5285414	0.75130128
C	-0.79746217	11.7914037	2.08192775
H	-1.83587103	11.6831656	2.3989005
C	0.16210767	12.1786865	3.02366902
C	1.53319001	12.3247147	2.66644393
C	1.59449264	15.421986	2.54174474
H	1.48471272	16.4725833	2.24913151
H	0.68581822	14.8912852	2.22896112
H	2.41908676	14.9866096	1.96621453
C	1.45332532	17.65486	7.03317559
H	0.74395103	18.3245819	6.53508809
H	2.37423465	18.2259586	7.20957678
H	1.04073342	17.3941403	8.01433684
H	15.0945327	6.23026326	-0.87298855

2·K$_1$ (as 2^{1-}, doublet, gasphase) E = -58927.18 eV

N	4.54645856	13.53190042	10.34854170
B	6.10771740	8.19277313	4.34826317
C	5.10738677	13.93590169	9.10582227
C	6.03729147	13.08512321	8.46045633
H	6.35555248	12.17355788	8.95936607
C	6.51159865	13.39028550	7.21000128
H	7.21579498	12.71656121	6.73545756
C	6.11967776	14.57726685	6.53078556
C	5.32843585	15.50481952	7.26135434
C	4.78509072	15.14096360	8.51675237
H	4.12340072	15.83799264	9.02671488
C	6.58997726	14.91437456	5.20343427
C	6.55946083	16.27917629	4.82888576
C	5.77464784	17.20264139	5.59642338
H	5.69993521	18.22788976	5.23883558
C	5.12830894	16.81952108	6.73020839
H	4.50955872	17.52056499	7.28616439
C	7.17575917	13.95475469	4.28404528
C	8.09136134	14.46270923	3.31623066
C	8.15154271	15.85199901	3.06007575
H	8.82169293	16.20524800	2.27828445
C	7.33409213	16.73285058	3.72692884

2·K$_2$ (as 2^{2-}, closed-shell S$_0$, gasphase) E = -58925.38 eV

N	4.48299573	13.56917080	10.33786862
B	6.14531835	8.21124612	4.37953811
C	5.05432897	13.99262701	9.09337058
C	6.00311795	13.16556990	8.44603985
H	6.35091366	12.26487371	8.94804205
C	6.44146028	13.46014266	7.17986279
H	7.14873335	12.79686920	6.69430715
C	6.00483476	14.63324115	6.48098046
C	5.17965203	15.54457582	7.22498700
C	4.67309809	15.17738613	8.48620027
H	3.98404250	15.85092748	8.99592206
C	6.41552075	14.94579588	5.15079156
C	6.30700867	16.30559477	4.71394288
C	5.46160870	17.19485729	5.47040521
H	5.29382078	18.19560725	5.07162895
C	4.88742433	16.82217099	6.64639999
H	4.23490705	17.50516419	7.18983132
C	7.04170244	13.97603137	4.23982478
C	7.98172407	14.53015012	3.28392741
C	7.95954074	15.88856754	2.99093649
H	8.63218165	16.26459348	2.21882139
C	7.05401454	16.76238862	3.61672882

H	7.31817009	17.78949800	3.47066822	H	6.98658947	17.80744611	3.32013409
C	6.85325152	12.56064368	4.25338368	C	6.75791547	12.60557591	4.21293316
C	7.68476207	11.65032123	3.52155376	C	7.61175678	11.68292553	3.47607518
C	8.73986451	12.19611438	2.70752642	C	8.68552123	12.26401708	2.69310226
H	9.37089134	11.50345046	2.15359159	H	9.34685367	11.58381423	2.15548623
C	8.89248902	13.53851207	2.56461857	C	8.83760249	13.60591327	2.57563682
H	9.63213636	13.94305410	1.87571317	H	9.60632235	14.01959467	1.92110781
C	5.67501969	12.00009564	4.84423091	C	5.60992428	11.99400968	4.84995312
H	4.93644778	12.66615247	5.28074129	H	4.85661458	12.64244401	5.29088607
C	5.44474165	10.65228664	4.84293281	C	5.42464816	10.64501363	4.86615221
H	4.52127749	10.27867988	5.28577928	H	4.51534994	10.25376860	5.32604566
C	6.34847294	9.69851614	4.25947125	C	6.33806027	9.69074025	4.26748190
C	7.44187490	10.27366423	3.56928571	C	7.41055401	10.32029300	3.53738482
H	8.13752327	9.61710968	3.04550480	H	8.12923662	9.67990958	3.02119122
C	5.07068764	7.56563924	5.38982173	C	5.12079151	7.55153704	5.42361187
C	4.12332542	6.59504731	4.96864826	C	4.22736509	6.52014989	5.01595761
C	3.28781515	5.95699581	5.88635890	C	3.41620188	5.85065241	5.93282194
H	2.57316650	5.21579088	5.52620304	H	2.74533804	5.06674725	5.57476870
C	3.35235485	6.22850745	7.25355357	C	3.44537655	6.14800255	7.29730717
C	4.27305453	7.18430295	7.67529927	C	4.30488353	7.16320894	7.70994048
H	4.36278734	7.40524012	8.73984837	H	4.36787637	7.40933029	8.77162393
C	5.11384671	7.85644061	6.77772062	C	5.12277863	7.86485257	6.81164400
C	3.98809997	6.20871723	3.51183362	C	4.12136622	6.10978008	3.56300692
H	3.99320863	7.09020206	2.86261548	H	4.11999883	6.98727232	2.90798518
H	3.05272116	5.66370207	3.34376807	H	3.19964835	5.54066117	3.38940698
H	4.81859311	5.57220558	3.18531458	H	4.97071656	5.48964843	3.25213032
C	2.44852484	5.51423236	8.22881767	C	2.57245211	5.39665452	8.27380657
H	2.25312287	4.48626165	7.90486885	H	2.50586003	4.33449920	8.00877624
H	1.47657244	6.01675178	8.31989876	H	1.54630176	5.78989698	8.29377592
H	2.89400776	5.47583447	9.22847323	H	2.96794831	5.46623466	9.29330004
C	6.08450229	8.85392470	7.37352425	C	6.03674166	8.91246207	7.40649331
H	7.02267836	8.89780339	6.81294520	H	6.97874442	8.97973590	6.85345028
H	6.30086768	8.59992523	8.41740003	H	6.23931558	8.69054567	8.46145695
H	5.67131095	9.87121372	7.36304537	H	5.59174445	9.91546833	7.36105958
C	6.93189811	7.19259691	3.41337124	C	6.99885951	7.19615060	3.47417469
C	6.92010491	7.29958067	2.00240765	C	7.01518817	7.26811563	2.05659966
C	7.61569629	6.37719562	1.21020803	C	7.72486704	6.32949214	1.29555679
H	7.57307064	6.47676551	0.12475732	H	7.70054235	6.40748769	0.20672135
C	8.35058532	5.33417329	1.76573719	C	8.44920062	5.29518341	1.88209113
C	8.36580075	5.22413089	3.15755263	C	8.43915980	5.21467348	3.27642029
H	8.93336648	4.41537060	3.62000259	H	9.00121261	4.41703263	3.76687260
C	7.67353833	6.12065351	3.97271586	C	7.73420797	6.13018039	4.06021244
C	6.14266252	8.38039308	1.28245940	C	6.24067451	8.32550057	1.30302563
H	5.81610480	8.02667894	0.29789502	H	5.98232504	7.97110009	0.29721372
H	6.75303461	9.27921022	1.13107786	H	6.81733361	9.25301515	1.20138498
H	5.26678778	8.70223239	1.85196442	H	5.32909891	8.60641691	1.83900197
C	9.12238526	4.36621864	0.90245548	C	9.23859380	4.31324295	1.05008873
H	8.99259930	3.33386084	1.24609232	H	9.12449472	3.28807216	1.42307127
H	10.19735465	4.58452160	0.92371354	H	10.31199709	4.54547365	1.06178468

H	8.79456075	4.41977212	-0.14079765
C	7.73822929	5.91669764	5.47063482
H	8.53011156	5.20577239	5.73070491
H	6.78982989	5.53285548	5.86452092
H	7.93644766	6.85910427	5.99238528
C	4.05370900	12.21208352	10.47912106
C	4.08897640	11.55757217	11.71925408
H	4.49185000	12.07583789	12.58491323
C	3.61058333	10.25906794	11.84196224
H	3.64258909	9.77413610	12.81624968
C	3.10894730	9.55521319	10.74105681
C	3.10001789	10.20650593	9.50617534
H	2.73502504	9.68132582	8.62546713
C	3.55003592	11.51816778	9.37234869
H	3.52608788	12.00024857	8.39934705
C	2.60681015	8.14126238	10.88868890
H	3.43060240	7.44227485	11.07937480
H	2.09911102	7.81023635	9.97851943
H	1.90287271	8.05479545	11.72405245
C	4.34078774	14.48556101	11.36738666
C	5.32575313	15.44778570	11.63896699
H	6.24124004	15.44436721	11.05429164
C	5.12841321	16.39644879	12.63315070
H	5.90765982	17.13227378	12.82387158
C	3.95867727	16.41905900	13.40301819
C	2.98739030	15.45576548	13.12849564
H	2.06282571	15.45036777	13.70235497
C	3.16330405	14.50716123	12.12252641
H	2.38687338	13.77523004	11.91977394
C	3.76566904	17.45304510	14.48439809
H	3.79508688	18.46869695	14.07391657
H	4.55162192	17.38520664	15.24522861
H	2.80222174	17.32274753	14.98618872

2·K$_2$ (as 2^{2-}, open-shell S$_0$, gasphase) E = -58925.46 eV

N	4.40211277	13.6397409	10.3584486
B	6.18525494	8.13889696	4.39144599
C	5.01684784	14.037056	9.12684807
C	5.98913227	13.207736	8.52962509
H	6.32878273	12.3220213	9.06400034
C	6.47208065	13.4729964	7.2707766
H	7.20885969	12.8075864	6.83484652
C	6.04338717	14.6247618	6.52179442
C	5.17743704	15.5431888	7.21802303
C	4.64094095	15.2105435	8.46810379
H	3.9345503	15.8934914	8.94011127
C	6.48382764	14.9124777	5.2029191
C	6.36622684	16.2626186	4.71961484
C	5.48452716	17.1574801	5.42167751

H	8.91054423	4.33014823	0.00458066
C	7.78348093	5.96041287	5.56299372
H	8.59628216	5.28214164	5.85055287
H	6.84264051	5.55522428	5.95492625
H	7.93683708	6.92468669	6.05944082
C	3.91468409	12.27956441	10.40348813
C	3.88016297	11.55478516	11.60717608
H	4.28484100	11.99998637	12.51155948
C	3.34563885	10.27260143	11.64543274
H	3.32988483	9.73591262	12.59363725
C	2.85493308	9.64609383	10.49431416
C	2.92782395	10.35759693	9.29508924
H	2.59585214	9.88576043	8.37201409
C	3.43387979	11.65380533	9.24305513
H	3.49133174	12.17364424	8.29166757
C	2.27341090	8.25604458	10.55079849
H	1.99791627	7.90959572	9.55070191
H	1.37689995	8.22314044	11.18300226
H	2.99178929	7.53604659	10.96199853
C	4.41984685	14.46717607	11.40926009
C	5.43860272	15.42271005	11.59067364
H	6.26589218	15.44207889	10.88774411
C	5.37990294	16.33151033	12.63717938
H	6.18635885	17.05500130	12.74988281
C	4.31736922	16.33990361	13.55061435
C	3.30534160	15.39854650	13.35958983
H	2.45349409	15.38500400	14.03822028
C	3.34218021	14.48363353	12.30958316
H	2.52784938	13.77787661	12.17762969
C	4.27609110	17.33382575	14.68504459
H	4.31219415	18.36606133	14.31662223
H	5.12530452	17.20245755	15.36707622
H	3.35845823	17.22146935	15.27192930

2·K$_2$ (as 2^{2-}, triplet, gasphase) E = -58925.42 eV

N	4.38090232	13.614365	10.3001468
B	6.17282843	8.15080657	4.46608732
C	5.05492136	14.023671	9.09738968
C	6.09201343	13.23816	8.59361362
H	6.41702944	12.3532779	9.13414765
C	6.63221349	13.553755	7.34057455
H	7.39281752	12.9029599	6.92053651
C	6.18490145	14.6484881	6.58812848
C	5.27108438	15.5712171	7.21152753
C	4.64948833	15.1747381	8.43175862
H	3.86832128	15.8003091	8.86042966
C	6.66121447	14.9369641	5.23235138
C	6.61705648	16.3188632	4.82223535
C	5.80143141	17.2301082	5.50647139

H	5.31893061	18.1483921	4.99704669
C	4.8812633	16.8039921	6.58704313
H	4.20672867	17.4922302	7.09575583
C	7.15507729	13.9437229	4.32376596
C	8.08123399	14.4661313	3.36882129
C	8.08568813	15.8425218	3.05714534
H	8.7640631	16.203638	2.28546212
C	7.15889133	16.7025207	3.6508359
H	7.08036249	17.7409048	3.32959206
C	6.87909262	12.5458501	4.31421284
C	7.73900941	11.636326	3.60678208
C	8.80527203	12.1946199	2.82463508
H	9.47464067	11.5102348	2.30348434
C	8.93824137	13.541867	2.67602847
H	9.70029926	13.9514887	2.01247108
C	5.72054232	11.9557342	4.92128754
H	4.97521811	12.6090389	5.3655271
C	5.51116678	10.6021588	4.91229728
H	4.59307567	10.216249	5.35796272
C	6.42258405	9.65650608	4.32571195
C	7.51694541	10.2524936	3.66071281
H	8.23794555	9.60753305	3.15338221
C	5.19841956	7.4858064	5.46086177
C	4.27573647	6.4672292	5.07976699
C	3.4821931	5.8121119	6.02209419
H	2.79044354	5.0373	5.68454795
C	3.5502198	6.11249391	7.3842194
C	4.43920604	7.11404853	7.77115613
H	4.53402036	7.36122882	8.83032678
C	5.24590462	7.79623051	6.85051816
C	4.11721592	6.0538869	3.63250344
H	4.09488417	6.92930747	2.97443249
H	3.18832156	5.48669853	3.4945706
H	4.95237708	5.43062135	3.29024184
C	2.69280069	5.37570635	8.38499306
H	2.62845514	4.30820053	8.14110151
H	1.6647525	5.7636146	8.41086221
H	3.10058466	5.46626197	9.39795849
C	6.2102365	8.81514616	7.41553865
H	7.15863865	8.81083348	6.86811108
H	6.40059372	8.6142899	8.47650257
H	5.821359	9.83833361	7.33746939
C	6.9652567	7.15999916	3.40045528
C	6.94630633	7.329611	1.98801685
C	7.60146687	6.42625152	1.14393424
H	7.5482898	6.58051542	0.06429107

H	5.75437141	18.2583493	5.14819337
C	5.05272936	16.8384562	6.62265449
H	4.38598832	17.5411223	7.1191805
C	7.24713254	13.969252	4.35351231
C	8.13140757	14.425684	3.32799824
C	8.2069346	15.8284861	3.04420222
H	8.86561766	16.1555102	2.23918616
C	7.43081155	16.7263692	3.71147227
H	7.43911556	17.7814853	3.43759425
C	6.95951952	12.5418309	4.37327932
C	7.78286158	11.6276129	3.65100226
C	8.80658662	12.1429649	2.7899052
H	9.43171566	11.4412098	2.24084416
C	8.91342062	13.494419	2.59317118
H	9.62193271	13.889099	1.86389214
C	5.80329342	11.9901139	4.99453436
H	5.08452236	12.6638039	5.45140939
C	5.55974139	10.6385617	5.00746936
H	4.64283033	10.2752288	5.47346941
C	6.44357646	9.67876169	4.41215332
C	7.53341754	10.2394387	3.73039727
H	8.22484857	9.57607368	3.20675523
C	5.24276837	7.49022455	5.57517839
C	4.30361831	6.47265645	5.22893237
C	3.58066326	5.7863434	6.20473342
H	2.8785922	5.00947636	5.89407743
C	3.73409427	6.05496446	7.56668553
C	4.62663733	7.0667085	7.91856057
H	4.77864535	7.29726753	8.97482365
C	5.36523441	7.77929556	6.96483452
C	4.05308629	6.09096545	3.78596227
H	4.00256129	6.97943849	3.14719323
H	3.11021122	5.53778038	3.69403926
H	4.85726335	5.46413441	3.38143524
C	2.96991121	5.26558838	8.60242659
H	3.01330622	4.1897753	8.39150162
H	1.90771414	5.54574942	8.63471165
H	3.38124824	5.42860238	9.60452468
C	6.34687948	8.80396962	7.48750252
H	7.2772015	8.79391065	6.90919148
H	6.57463948	8.61250261	8.5424906
H	5.95504145	9.82577771	7.41405954
C	6.87879051	7.18798751	3.40873024
C	6.78895849	7.39966052	2.00446215
C	7.38010256	6.51007848	1.10156508
H	7.27297655	6.69688446	0.031165

C	8.30479027	5.32741184	1.63530553
C	8.32258904	5.14580497	3.01914174
H	8.86418851	4.29397573	3.43559457
C	7.67316748	6.02640217	3.88772595
C	6.17403617	8.45433528	1.33754296
H	5.96374409	8.22046889	0.28673881
H	6.72276223	9.40269341	1.37616925
H	5.23013486	8.63945471	1.86169809
C	9.04341016	4.38918236	0.71182356
H	9.03651085	3.36247258	1.09678625
H	10.0945507	4.684473	0.59015319
H	8.59050709	4.37847285	-0.28644271
C	7.75300202	5.73903374	5.37098276
H	8.53594076	4.99981275	5.58051171
H	6.80312393	5.35324828	5.76122685
H	7.96959411	6.65241039	5.93599562
C	3.86303772	12.3392219	10.4404849
C	3.83780929	11.6269423	11.6522998
H	4.22003508	12.0941517	12.555322
C	3.34629197	10.3274245	11.6991075
H	3.34071107	9.80000805	12.6527371
C	2.88952443	9.672094	10.5506422
C	2.94767959	10.3745419	9.34430415
H	2.63854978	9.88206301	8.42411889
C	3.41238952	11.6848968	9.28303918
H	3.46575855	12.1979568	8.32777662
C	2.36172553	8.26102604	10.6129175
H	2.12133468	7.89316636	9.61137859
H	1.453822	8.19845676	11.226544
H	3.09907331	7.57436083	11.0469803
C	4.30987116	14.5531011	11.4125088
C	5.29575757	15.5473932	11.5752451
H	6.12241016	15.5800694	10.8722938
C	5.20577453	16.4732162	12.6048052
H	5.98859817	17.2244419	12.7038063
C	4.14277716	16.4663098	13.5170599
C	3.16211536	15.4876797	13.3436744
H	2.31085257	15.4584568	14.0227707
C	3.23029912	14.5540117	12.3127406
H	2.43927613	13.8196458	12.1957096
C	4.05856958	17.4902897	14.6220127
H	3.92219517	18.5036642	14.2241395
H	4.97038823	17.5012924	15.2315772
H	3.21526297	17.2800095	15.288378

2·K_2 (as 2^{2-}, closed-shell S_0, SMD=Et_2O) E = -58930.42 eV

C	8.08702256	5.38457637	1.52376111
C	8.1751256	5.16278837	2.89860621
H	8.72169865	4.28985531	3.26111623
C	7.59061556	6.02929042	3.82598728
C	6.00712943	8.55831761	1.4293251
H	5.7384264	8.3612746	0.38448403
H	6.5770265	9.4940947	1.46760111
H	5.09391337	8.74361161	2.00569014
C	8.75720761	4.46149332	0.53515104
H	8.78930861	3.43168924	0.90998707
H	9.79263068	4.76761734	0.33278202
H	8.22650458	4.45533032	-0.42410003
C	7.74669655	5.69788438	5.29393339
H	8.52687559	4.94046036	5.43838438
H	6.8133905	5.31625339	5.72627342
H	8.01033657	6.59082649	5.87167043
C	3.85278228	12.3092459	10.3512098
C	3.75860427	11.6004318	11.5594278
H	4.0906683	12.0682859	12.4816829
C	3.26179523	10.3003698	11.5795408
H	3.20160423	9.77316569	12.5313209
C	2.86830521	9.64745667	10.4082298
C	2.99821621	10.3473428	9.20504767
H	2.7404832	9.85330271	8.27002057
C	3.46722625	11.6567929	9.16959967
H	3.57046826	12.1728079	8.21985362
C	2.31637717	8.24473262	10.4391478
H	2.25354416	7.83294758	9.42798369
H	1.31186609	8.21899259	10.8814348
H	2.95284121	7.57557153	11.0307318
C	4.22441131	14.5312591	11.3437378
C	5.20118337	15.5237741	11.5571968
H	6.06432746	15.5554231	10.8994658
C	5.05462836	16.4525652	12.5781619
H	5.82747539	17.2078472	12.7155569
C	3.94577028	16.4416572	13.433904
C	2.97790422	15.4613861	13.212698
H	2.09238915	15.4317771	13.8463
C	3.10146822	14.52697	12.1855619
H	2.31873317	13.791884	12.0253989
C	3.81080527	17.4548933	14.5441041
H	3.85487528	18.4806663	14.159102
H	4.61329133	17.3510742	15.2850901
H	2.85708321	17.3366183	15.0692721

2·K_2 (as 2^{2-}, open-shell S_0, SMD=Et_2O) E = -58930.46 eV

N	4.48299573	13.5691708	10.3378686	N	4.4546703	13.6094811	10.3586775
B	6.14531835	8.21124612	4.37953811	B	6.13857632	8.15746277	4.35162831
C	5.05432897	13.992627	9.09337058	C	5.0198118	14.0101626	9.10953096
C	6.00311795	13.1655699	8.44603985	C	5.97743401	13.1783711	8.4824846
H	6.35091366	12.2648737	8.94804205	H	6.33070036	12.2917706	9.00489918
C	6.44146028	13.4601427	7.17986279	C	6.43836751	13.4586733	7.21978053
H	7.14873335	12.7968692	6.69430715	H	7.16628404	12.7938739	6.76710427
C	6.00483476	14.6332412	6.48098046	C	6.01019257	14.6211531	6.49834287
C	5.17965203	15.5445758	7.224987	C	5.17048536	15.5374783	7.21939698
C	4.67309809	15.1773861	8.48620027	C	4.64984329	15.1891298	8.48180461
H	3.9840425	15.8509275	8.99592206	H	3.96004446	15.8742271	8.97389638
C	6.41552075	14.9457959	5.15079156	C	6.43656613	14.9217562	5.16861848
C	6.30700867	16.3055948	4.71394288	C	6.32800405	16.2763023	4.7140172
C	5.4616087	17.1948573	5.47040521	C	5.46720094	17.1721163	5.44811103
H	5.29382078	18.1956073	5.07162895	H	5.29805393	18.1673519	5.03798031
C	4.88742433	16.822171	6.64639999	C	4.87949547	16.8079917	6.62001315
H	4.23490705	17.5051642	7.18983132	H	4.21742305	17.4945116	7.14580885
C	7.04170244	13.9760314	4.23982478	C	7.07629206	13.9468005	4.26686841
C	7.98172407	14.5301501	3.28392741	C	8.01833615	14.4908903	3.31324543
C	7.95954074	15.8885675	2.99093649	C	8.00935506	15.8540905	3.01371732
H	8.63218165	16.2645935	2.21882139	H	8.69092609	16.2253787	2.24854677
C	7.05401454	16.7623886	3.61672882	C	7.09173472	16.7268828	3.62390335
H	6.98658947	17.8074461	3.32013409	H	7.02265671	17.7687747	3.31798979
C	6.75791547	12.6055759	4.21293316	C	6.78796793	12.5685581	4.23848079
C	7.61175678	11.6829255	3.47607518	C	7.63688449	11.648141	3.5022799
C	8.68552123	12.2640171	2.69310226	C	8.71717027	12.2196834	2.7270489
H	9.34685367	11.5838142	2.15548623	H	9.38093004	11.5391872	2.19349696
C	8.83760249	13.6059133	2.57563682	C	8.87539723	13.5634708	2.61140955
H	9.60632235	14.0195947	1.92110781	H	9.65120436	13.9717218	1.96324353
C	5.60992428	11.9940097	4.84995312	C	5.64104138	11.9585417	4.87441357
H	4.85661458	12.642444	5.29088607	H	4.89475193	12.6015458	5.33335079
C	5.42464816	10.6450136	4.86615221	C	5.44558188	10.6063324	4.87805825
H	4.51534994	10.2537686	5.32604566	H	4.53773207	10.2239231	5.34836657
C	6.33806027	9.69074025	4.2674819	C	6.34346989	9.6533243	4.26474668
C	7.41055401	10.320293	3.53738482	C	7.41984874	10.278332	3.54888675
H	8.12923662	9.67990958	3.02119122	H	8.12997683	9.64089408	3.01761421
C	5.12079151	7.55153704	5.42361187	C	5.12229515	7.49097556	5.39905545
C	4.22736509	6.52014989	5.01595761	C	4.20173576	6.48519456	4.98770102
C	3.41620188	5.85065241	5.93282194	C	3.37387299	5.83290311	5.90523921
H	2.74533804	5.06674725	5.5747687	H	2.67873404	5.07276339	5.54512943
C	3.44537655	6.14800255	7.29730717	C	3.41240476	6.12209112	7.27135367
C	4.30488353	7.16320894	7.70994048	C	4.3100624	7.10334534	7.68825543
H	4.36787637	7.40933029	8.77162393	H	4.38039346	7.34155866	8.75060855
C	5.12277863	7.86485257	6.811644	C	5.14589707	7.78577185	6.79163777
C	4.12136622	6.10978008	3.56300692	C	4.08042494	6.07307899	3.53671194
H	4.11999883	6.98727232	2.90798518	H	4.12190262	6.93997467	2.8697198

H	3.19964835	5.54066117	3.38940698
H	4.97071656	5.48964843	3.25213032
C	2.57245211	5.39665452	8.27380657
H	2.50586003	4.3344992	8.00877624
H	1.54630176	5.78989698	8.29377592
H	2.96794831	5.46623466	9.29330004
C	6.03674166	8.91246207	7.40649331
H	6.97874442	8.9797359	6.85345028
H	6.23931558	8.69054567	8.46145695
H	5.59174445	9.91546833	7.36105958
C	6.99885951	7.1961506	3.47417469
C	7.01518817	7.26811563	2.05659966
C	7.72486704	6.32949214	1.29555679
H	7.70054235	6.40748769	0.20672135
C	8.44920062	5.29518341	1.88209113
C	8.4391598	5.21467348	3.27642029
H	9.00121261	4.41703263	3.7668726
C	7.73420797	6.13018039	4.06021244
C	6.24067451	8.32550057	1.30302563
H	5.98232504	7.97110009	0.29721372
H	6.81733361	9.25301515	1.20138498
H	5.32909891	8.60641691	1.83900197
C	9.2385938	4.31324295	1.05008873
H	9.12449472	3.28807216	1.42307127
H	10.3119971	4.54547365	1.06178468
H	8.91054423	4.33014823	0.00458066
C	7.78348093	5.96041287	5.56299372
H	8.59628216	5.28214164	5.85055287
H	6.84264051	5.55522428	5.95492625
H	7.93683708	6.92468669	6.05944082
C	3.91468409	12.2795644	10.4034881
C	3.88016297	11.5547852	11.6071761
H	4.284841	11.9999864	12.5115595
C	3.34563885	10.2726014	11.6454327
H	3.32988483	9.73591262	12.5936373
C	2.85493308	9.64609383	10.4943142
C	2.92782395	10.3575969	9.29508924
H	2.59585214	9.88576043	8.37201409
C	3.43387979	11.6538053	9.24305513
H	3.49133174	12.1736442	8.29166757
C	2.2734109	8.25604458	10.5507985
H	1.99791627	7.90959572	9.55070191
H	1.37689995	8.22314044	11.1830023
H	2.99178929	7.53604659	10.9619985
C	4.41984685	14.4671761	11.4092601
C	5.43860272	15.4227101	11.5906736

H	3.1340674	5.54879737	3.36227365
H	4.89457527	5.40167567	3.23670482
C	2.50625831	5.40802289	8.24438331
H	2.3768605	4.35582375	7.9668798
H	1.50662313	5.86132898	8.26931719
H	2.90897957	5.44171603	9.26226697
C	6.09043854	8.79984892	7.39752439
H	7.05792871	8.8178201	6.88561605
H	6.25218342	8.58541619	8.46038955
H	5.69141514	9.81981346	7.32939251
C	6.98778044	7.17084365	3.4130983
C	6.98908597	7.27954353	1.99688302
C	7.72097102	6.38294005	1.20501151
H	7.68789485	6.48964764	0.1197851
C	8.48027772	5.35390499	1.75829427
C	8.47786928	5.2332195	3.15040981
H	9.0645528	4.43818139	3.61353189
C	7.75297984	6.10705206	3.9654128
C	6.18262826	8.3335628	1.27099236
H	5.95373587	8.00977697	0.24908715
H	6.72498827	9.28548721	1.2071906
H	5.24532485	8.55743288	1.79075255
C	9.29423546	4.41953763	0.89716686
H	9.17888895	3.3770964	1.21539415
H	10.3638097	4.65896993	0.95172741
H	8.99483973	4.48752967	-0.15391119
C	7.81520326	5.882327	5.46076863
H	8.65021347	5.22170082	5.71990737
H	6.89427064	5.42131439	5.8392552
H	7.94090582	6.82546389	6.00316166
C	3.91778145	12.3097527	10.4675036
C	3.93229544	11.6160917	11.6901015
H	4.34912612	12.0923374	12.5732526
C	3.42648473	10.3241475	11.7761222
H	3.45062839	9.81233179	12.737162
C	2.90943086	9.65990716	10.6561401
C	2.918534	10.3483336	9.4405689
H	2.54025269	9.86015666	8.54410084
C	3.40185219	11.6518806	9.34122439
H	3.39736082	12.1541781	8.37809566
C	2.37392835	8.25450689	10.7642969
H	1.98489704	7.91099212	9.80123362
H	1.56212447	8.19205123	11.4988989
H	3.15440923	7.55221598	11.0817971
C	4.37658773	14.5347975	11.4190913
C	5.42402542	15.4442717	11.641293

H	6.26589218	15.4420789	10.8877441
C	5.37990294	16.3315103	12.6371794
H	6.18635885	17.0550013	12.7498828
C	4.31736922	16.3399036	13.5506144
C	3.3053416	15.3985465	13.3595898
H	2.45349409	15.385004	14.0382203
C	3.34218021	14.4836335	12.3095832
H	2.52784938	13.7778766	12.1776297
C	4.2760911	17.3338258	14.6850446
H	4.31219415	18.3660613	14.3166222
H	5.12530452	17.2024576	15.3670762
H	3.35845823	17.2214694	15.2719293

H	6.29577472	15.4187378	10.9933361
C	5.34827825	16.370186	12.6746744
H	6.17576965	17.0618783	12.8241142
C	4.24106043	16.4268204	13.5317442
C	3.2027025	15.5213877	13.3009621
H	2.3204592	15.5456114	13.937928
C	3.25696656	14.5960294	12.2595233
H	2.42365158	13.9182739	12.0950109
C	4.18334738	17.4308741	14.6554064
H	4.29249466	18.4549365	14.2804114
H	4.9883568	17.2627751	15.3806418
H	3.23136389	17.3688188	15.1915239

2·K_2 (as 2^{2-}, triplet, SMD=Et_2O) E = -58930.34 eV

N	4.38090232	13.614365	10.3001468
B	6.17282843	8.15080657	4.46608732
C	5.05492136	14.023671	9.09738968
C	6.09201343	13.23816	8.59361362
H	6.41702944	12.3532779	9.13414765
C	6.63221349	13.553755	7.34057455
H	7.39281752	12.9029599	6.92053651
C	6.18490145	14.6484881	6.58812848
C	5.27108438	15.5712171	7.21152753
C	4.64948833	15.1747381	8.43175862
H	3.86832128	15.8003091	8.86042966
C	6.66121447	14.9369641	5.23235138
C	6.61705648	16.3188632	4.82223535
C	5.80143141	17.2301082	5.50647139
H	5.75437141	18.2583493	5.14819337
C	5.05272936	16.8384562	6.62265449
H	4.38598832	17.5411223	7.1191805
C	7.24713254	13.969252	4.35351231
C	8.13140757	14.425684	3.32799824
C	8.2069346	15.8284861	3.04420222
H	8.86561766	16.1555102	2.23918616
C	7.43081155	16.7263692	3.71147227
H	7.43911556	17.7814853	3.43759425
C	6.95951952	12.5418309	4.37327932
C	7.78286158	11.6276129	3.65100226
C	8.80658662	12.1429649	2.7899052
H	9.43171566	11.4412098	2.24084416
C	8.91342062	13.494419	2.59317118
H	9.62193271	13.889099	1.86389214
C	5.80329342	11.9901139	4.99453436
H	5.08452236	12.6638039	5.45140939
C	5.55974139	10.6385617	5.00746936

H	4.64283033	10.2752288	5.47346941
C	6.44357646	9.67876169	4.41215332
C	7.53341754	10.2394387	3.73039727
H	8.22484857	9.57607368	3.20675523
C	5.24276837	7.49022455	5.57517839
C	4.30361831	6.47265645	5.22893237
C	3.58066326	5.7863434	6.20473342
H	2.8785922	5.00947636	5.89407743
C	3.73409427	6.05496446	7.56668553
C	4.62663733	7.0667085	7.91856057
H	4.77864535	7.29726753	8.97482365
C	5.36523441	7.77929556	6.96483452
C	4.05308629	6.09096545	3.78596227
H	4.00256129	6.97943849	3.14719323
H	3.11021122	5.53778038	3.69403926
H	4.85726335	5.46413441	3.38143524
C	2.96991121	5.26558838	8.60242659
H	3.01330622	4.1897753	8.39150162
H	1.90771414	5.54574942	8.63471165
H	3.38124824	5.42860238	9.60452468
C	6.34687948	8.80396962	7.48750252
H	7.2772015	8.79391065	6.90919148
H	6.57463948	8.61250261	8.5424906
H	5.95504145	9.82577771	7.41405954
C	6.87879051	7.18798751	3.40873024
C	6.78895849	7.39966052	2.00446215
C	7.38010256	6.51007848	1.10156508
H	7.27297655	6.69688446	0.031165
C	8.08702256	5.38457637	1.52376111
C	8.1751256	5.16278837	2.89860621
H	8.72169865	4.28985531	3.26111623
C	7.59061556	6.02929042	3.82598728
C	6.00712943	8.55831761	1.4293251
H	5.7384264	8.3612746	0.38448403
H	6.5770265	9.4940947	1.46760111
H	5.09391337	8.74361161	2.00569014
C	8.75720761	4.46149332	0.53515104
H	8.78930861	3.43168924	0.90998707
H	9.79263068	4.76761734	0.33278202
H	8.22650458	4.45533032	-0.42410003
C	7.74669655	5.69788438	5.29393339
H	8.52687559	4.94046036	5.43838438
H	6.8133905	5.31625339	5.72627342
H	8.01033657	6.59082649	5.87167043
C	3.85278228	12.3092459	10.3512098
C	3.75860427	11.6004318	11.5594278

H	4.0906683	12.0682859	12.4816829
C	3.26179523	10.3003698	11.5795408
H	3.20160423	9.77316569	12.5313209
C	2.86830521	9.64745667	10.4082298
C	2.99821621	10.3473428	9.20504767
H	2.7404832	9.85330271	8.27002057
C	3.46722625	11.6567929	9.16959967
H	3.57046826	12.1728079	8.21985362
C	2.31637717	8.24473262	10.4391478
H	2.25354416	7.83294758	9.42798369
H	1.31186609	8.21899259	10.8814348
H	2.95284121	7.57557153	11.0307318
C	4.22441131	14.5312591	11.3437378
C	5.20118337	15.5237741	11.5571968
H	6.06432746	15.5554231	10.8994658
C	5.05462836	16.4525652	12.5781619
H	5.82747539	17.2078472	12.7155569
C	3.94577028	16.4416572	13.433904
C	2.97790422	15.4613861	13.212698
H	2.09238915	15.4317771	13.8463
C	3.10146822	14.52697	12.1855619
H	2.31873317	13.791884	12.0253989
C	3.81080527	17.4548933	14.5441041
H	3.85487528	18.4806663	14.159102
H	4.61329133	17.3510742	15.2850901
H	2.85708321	17.3366183	15.0692721
